# Comparison of Antifungal Prophylaxis Drugs in Patients With Hematological Disease or Undergoing Hematopoietic Stem Cell Transplantation: A Systematic Review and Network Meta-analysis

**DOI:** 10.1001/jamanetworkopen.2020.17652

**Published:** 2020-10-08

**Authors:** Jing Wang, Min Zhou, Jing-Yan Xu, Rong-Fu Zhou, Bing Chen, Yuan Wan

**Affiliations:** 1 Department of Hematology, Affiliated Drum Tower Hospital of Nanjing University Medical School, Nanjing, China; 2 The Pq Laboratory of Micro/Nano BiomeDx, Department of Biomedical Engineering, Binghamton University – SUNY, Binghamton, New York

## Abstract

**Importance:**

Several antifungal drugs are available for antifungal prophylaxis in patients with hematological disease or who are undergoing hematopoietic stem cell transplantation (HSCT).

**Objective:**

To summarize the evidence on the efficacy and adverse effects of antifungal agents using an integrated comparison.

**Data Sources:**

Medline, EMBASE, and the Cochrane Central Register of Controlled Clinical Trials were searched to collect all relevant evidence published in randomized clinical trials that assessed antifungal prophylaxis in patients with hematological disease. Sources were search from inception up to October 2019.

**Study Selection:**

Studies that compared any antifungal agent with a placebo, no antifungal agent, or another antifungal agent among patients with hematological disease or undergoing HSCT were included. Of 39 709 studies identified, 69 met the criteria for inclusion.

**Data Extraction and Synthesis:**

The outcome from each study was estimated using the relative risk (RR) with 95% CIs. The Mantel–Haenszel random-effects model was used. The reliability and validity of the networks were estimated by addressing inconsistencies in the evidence from comparative studies of different treatments. Data were analyzed from December 2019 to February 2020. Reporting followed the Preferred Reporting Items for Systematic Reviews and Meta-analyses for Network Meta-analysis (PRISMA-NMA) guideline.

**Main Outcomes and Measures:**

The primary outcomes were invasive fungal infections (IFIs) and mortality. The secondary outcomes were fungal infections, proven IFIs, invasive candidiasis, invasive aspergillosis, fungi-related death, and withdrawal owing to adverse effects of the drug.

**Results:**

We identified 69 randomized clinical trials that reported comparisons of 12 treatments with at total of 14 789 patients. Posaconazole was the treatment associated with the best probability of success against IFIs (surface under the cumulative ranking curve, 86.7%; mean rank, 2.5). Posaconazole treatment was associated with a significant reduction in IFIs (RR, 0.57; 95% CI, 0.42-0.79) and invasive aspergillosis (RR, 0.36; 95% CI, 0.15-0.85) compared with placebo. Voriconazole was associated with a significant reduction in invasive candidiasis (RR, 0.15; 95% CI, 0.09-0.26) compared with placebo. However, posaconazole was associated with a higher incidence of withdrawal because of the adverse effects of the drug (surface under the cumulative ranking curve, 17.5%; mean rank, 9.2). In subgroup analyses considering efficacy and tolerance, voriconazole might be the best choice for patients undergoing HSCT, especially allogenic HSCT; however, posaconazole was ranked as the best choice for patients with acute myeloid leukemia or myelodysplastic syndrome.

**Conclusions and Relevance:**

These findings suggest that voriconazole may be the best prophylaxis option for patients undergoing HSCT, and posaconazole may be the best prophylaxis option for patients with acute myeloid leukemia or myelodysplastic syndrome.

## Key Points

**Question:**

What primary antifungal prophylaxis drugs for patients with hematological disease or undergoing hematopoietic stem cell transplantation perform best in randomized clinical trials?

**Findings:**

In this systematic review and network meta-analysis of 69 randomized clinical trials that performed comparisons of individual antifungal agents in 14 789 patients, voriconazole was recommended for patients undergoing HSCT and posaconazole was recommended for patients with acute myeloid leukemia or myelodysplastic syndrome.

**Meaning:**

These findings may help clinicians to make antifungal prophylaxis treatment decisions.

## Teaser

This systematic review and network meta-analsysis compares antifungal prophylaxis drugs for patients with hematological disease or who are undergoing hematopoietic stem cell transplantation (HSCT).

## Introduction

Invasive fungal infections (IFIs) have emerged as important causes of morbidity and mortality in patients receiving myelosuppressive chemotherapy, immunosuppressive therapy, or hematopoietic stem cell transplantation (HSCT). Because of the difficulty in obtaining a timely diagnosis as well as the high morbidity and mortality associated with IFIs, antifungal prophylaxis remains a high priority in these populations at high risk of IFIs.^1^ Over the past decade, clinical benefits from antifungal prophylaxis have been demonstrated.^2,3,4^ However, there is no clear consensus on antifungal prophylaxis treatment between different centers and groups, particularly in the choice of the antifungal prophylaxis agents. Conventional pairwise meta-analyses based on a direct comparison are relatively limited and difficult to use to investigate antifungal prophylaxis agents. We performed a systematic review and network meta-analysis^5^ to gain a better understanding of the outcomes associated with and tolerance to current antifungal agents.

## Methods

### Protocol and Registration

This systematic review was conducted in accordance with the Preferred Reporting Items for Systematic Reviews and Meta-analyses for Network Meta-analysis (PRISMA-NMA) reporting guideline.^6^ This protocol has been registered at PROSPERO under registration number CRD42020161748.

### Literature Search

Medline, EMBASE, and the Cochrane Central Register of Controlled Clinical Trials were searched to collect all published evidence from randomized clinical trials from inception to October 2019 that assessed primary antifungal prophylaxis in patients with hematological disease or undergoing HSCT. The search strategy is detailed in the eAppendix in the Supplement. The reference lists from all included studies and reviews were screened to identify potentially relevant evidence.

### Study Inclusion Criteria

All available randomized clinical trials that aimed to compare any antifungal agent with a placebo, no antifungal agent, or another antifungal agent for prophylaxis in patients with hematological disease or undergoing HSCT were included. In this analysis, we assumed that there was no difference between placebo and no antifungal agent.

### Data Extraction

From each relevant study, the following data were extracted: authors’ names, year of publication, number of patients, age, use and dosage of drugs, categories of disease. Extracted outcomes included (1) incidence of fungal infections (superficial and IFI); (2) incidence of IFIs (possible, probable, and proven IFIs); (3) incidence of proven IFIs (positive histological results on biopsy from deep tissue); (4) incidence of invasive candidiasis; (5) incidence of invasive aspergillosis; (6) fungi-related death; and (7) withdrawal because of adverse effects of the drug.

### Quality Assessment

Two of us (M.Z. and J.-Y.X.) independently participated in the quality assessment, and disagreements were resolved by a third reviewer (B.C.) until consensus was obtained. The quality of the evidence was assessed using the revised tool for risk of bias in randomized trials.^7^

### Statistical Analysis

We compared different agents through network meta-analyses performed under a frequentist framework using a random-effects model. The analysis was performed using the *network* and *mvmeta* packages in Stata statistical software version 14.0 (StateCorp).^8,9^ We estimated the outcome from each study using the relative risk (RR) with 95% CIs. A 95% CI of an RR not covering 1 indicated a statistically significant association. Forest plots and league tables were used to visually present the results of the network meta-analysis. For each outcome, the surface under the cumulative ranking curve (SUCRA) was used to separately rank each agent.^10^ The larger the SUCRA value, the better the rank. The reliability and validity of the networks were estimated by addressing the inconsistencies and heterogeneity in the evidence from comparative studies of different treatments.^11^ The overall and loop inconsistencies were evaluated.^8,12^ Heterogeneity was estimated by the restricted maximum likelihood method. A τ^2^ value less than 0.1 indicated a very low level of heterogeneity, and a τ^2^ value from 0.1 to 0.5 indicated a reasonable level; a τ^2^ value greater than 0.5 was considered to indicate high heterogeneity.^13^ Additional subgroup analyses were performed restricted to data from different patient populations. Small-study effects were described with a funnel plot. Each funnel plot was tested using the Begg test to assess the small-study effects. A 2-sided *P* < .05 was considered statistically significant. Data were analyzed from December 2019 to February 2020.

## Results

### Characteristics of the Studies

The flowchart of study selection for this network meta-analysis is shown in eFigure 1 in the Supplement. In total, 69 trials with 14 789 patients were included,^14,15,16,17,18,19,20,21,22,23,24,25,26,27,28,29,30,31,32,33,34,35,36,37,38,39,40,41,42,43,44,45,46,47,48,49,50,51,52,53,54,55,56,57,58,59,60,61,62,63,64,65,66,67,68,69,70,71,72,73,74,75,76,77,78,79,80,81,82^ including 12 groups: placebo, polyene, conventional amphotericin B, liposomal amphotericin B, miconazole, ketoconazole, fluconazole, itraconazole, voriconazole, posaconazole, caspofungin, and micafungin. The basic characteristics of the included studies are summarized in Table 1. The randomization process and selection of the reported results were not reported clearly in most trials (eFigure 2 in the Supplement).

**Table 1.** Basic Characteristics of Included Studies

Source	Country	Population	Interventions		Patients, No.	Mean age, (range), y	RoB2
			A	B			
Akiyama et al,^14^ 1993	Japan	Patients with hematological malignant neoplasms receiving chemotherapy	FLCZ 200 mg orally every d	AMB 800 mg orally 3×/d	130	43.32 (15-67)	2
Annaloro et al,^15^ 1995	Italy	Bone marrow transplant recipients (allogeneic and autologous HSCT)	FLCZ 300 mg orally every d	ITCZ 400 mg po/IV every d	59	33.62 (13-56)	2
Behre et al,^16^ 1995	Germany	Patients with hematological diseases receiving chemotherapy or undergoing HSCT	AMB 10 mg INH 2×/d	Placebo	115	43.00 (18-81)	2
Benhamou et al,^17^ 1991	France	Bone marrow transplant recipients (allogeneic and autologous HSCT)	KTCZ 200-600 mg orally every d	Placebo	125	7.00 (NA)	2
Bodey et al,^18^ 1994	United States	Patients with acute leukemia receiving chemotherapy	FLCZ 400 mg orally every d	AMB 0.5 mg/kg IV 3×/wk	77	46.47 (17-80)	2
Boogaerts et al,^19^ 2001	Finland	Patients with hematological diseases receiving chemotherapy or HSCT	ITCZ 400 mg orally 2×/d	AMB 500 mg orally 3×/d Nystatin 2 mIU orally 4×/d	277	46.92 (NA)	2
Brincker,^20^ 1978	Denmark	Patients with hematological malignant neoplasms receiving chemotherapy	MICZ 500 mg orally 4×/d	Placebo	30	NA	2
Brincker,^21^ 1983	Denmark	Patients with acute leukemia receiving chemotherapy	KTCZ 400 mg orally every d	Placebo	38	58.50 (NA)	2
Chaftari et al,^22^ 2012	United States	Hematopoietic stem cell transplant recipients (allogeneic HSCT)	POCZ 200 mg orally 3×/d	AMBL 7.5 mg/kg IV 1×/wk	40	55.48 (20-69)	2
Chandrasekar and Gatney,^23^ 1994	United States	Patients with acute leukemia receiving chemotherapy or HSCT	FLCZ 400 mg orally every d	Placebo	46	38.00 (NA)	3
Choi et al,^24^ 2005	Korea	Hematopoietic stem cell transplant recipients (allogeneic HSCT)	FLCZ 200 mg orally every d	ITCZ 200 mg orally every d	78	34.43 (18-56)	3
Cornely et al,^25^ 2007	International	Patients with AML or MDS receiving chemotherapy	POCZ 200 mg orally 3×/d	FLCZ 400 mg orally every d or ITCZ 200 mg orally 2×/d	602	49.44 (13-82)	2
Donnelly et al,^26^ 1984	UK	Patients with acute leukemia receiving chemotherapy or HSCT	KTCZ 400 mg orally every d	AMB 100 mg orally 4×/d	36	37.38 (13-63)	2
Egger et al,^27^ 1995	Switzerland	Patients with hematological diseases receiving chemotherapy or HSCT	FLCZ 400 mg orally every d	Polyene, nystatin 8 mIU orally 3×/d	89	38.42 (14-73)	3
Ellis et al,^28^ 1994	Saudi Arabia	Patients with hematological malignancies receiving chemotherapy or HSCT	FLCZ 200 mg orally every d	Polyene, clotrimazole 10 mg 2×/d	90	23.33 (12-70)	2
Epstein et al,^29^ 2018	United States	Patients with hematological malignant neoplasms receiving chemotherapy	POCZ 200 mg orally 3×/d	MCFG 100 mg IV every d	113	60.02 (26-75)	2
Estey et al,^30^ 1984	United States	Patients with acute leukemia receiving chemotherapy	KTCZ 200 mg orally 2×/d	Placebo	70	37.22 (16-78)	3
Fisher et al,^31^ 2019	United States	Patients with acute myeloid leukemia receiving chemotherapy	FLCZ 400 mg orally every d	CASP 50 mg IV every d	510	9.49 (0-26)	2
Glasmache et al,^32^ 2006	Germany	Patients with hematological malignancies receiving chemotherapy or HSCT	FLCZ 400 mg orally every d	ITCZ 2.5mg/kg orally 2×/d	494	48.94 (NA)	1
Goodman et al,^33^ 1992	United States	Bone marrow transplant recipients (allogeneic and autologous HSCT)	FLCZ 400 mg orally every d	Placebo	356	NA	2
Hansen et al,^34^ 1987	United States	Patients with hematological malignant neoplasms receiving chemotherapy or HSCT	KTCZ 400 mg orally every d	Placebo	56	NA	2
Harousseau et al,^35^ 2000	International	Patients with hematological malignant neoplasms receiving chemotherapy or HSCT	ITCZ 2.5 mg/kg orally 2×/d	AMB 500 mg orally 4×/d	557	48.74 (15-82)	1
Hayashi et al,^36^ 2014	Japan	Patients with grade II-IV acute GVHD or chronic GVHD receiving corticosteroid treatment	ITCZ 2.5 mg/kg orally 2×/d	VOCZ 200 mg orally 2×/d	66	NA	2
Hiemenz et al,^37^ 2005	United States	Hematopoietic stem cell transplant recipients (allogeneic and autologous HSCT)	FLCZ 400 mg IV every d	MCFG 5 mg/kg IV 2×/d	74	43.24 (19-65)	3
Hiramatsu et al,^38^ 2008	Japan	Hematopoietic stem cell transplant recipients (allogeneic and autologous HSCT)	FLCZ 400 mg IV every d	MCFG 150 mg IV every d	100	46.90 (16-67)	2
Huang et al,^39^ 2012	China	Hematopoietic stem cell transplant recipients (allogeneic and autologous HSCT)	ITCZ 5 mg/kg orally every d	MCFG 50 mg IV every d	283	32.72 (18-70)	3
Huijgens et al,^40^ 1999	Netherlands	Patients with hematological malignant neoplasms receiving chemotherapy or HSCT	FLCZ 50 mg orally 2×/d	ITCZ 100 mg orally 2×/d	202	45.15 (NA)	2
Ito et al,^41^ 2007	Japan	Patients with AML or MDS receiving chemotherapy	FLCZ 200 mg orally every d	ITCZ 200 mg orally every d	218	55.46 (16-80)	3
Kaptan et al,^42^ 2003	Turkey	Patients with acute leukemia receiving chemotherapy	ITCZ 200 mg orally 2×/d	Placebo	97	35.58 (20-73)	3
Kelsey et al,^43^ 1999	International	Hematopoietic stem cell transplant recipients (allogeneic and autologous HSCT)	AMBL 2 mg/kg IV 3×/wk	Placebo	161	39.92 (15-65)	1
Kern et al,^44^ 1998	International	Patients with AML receiving chemotherapy	FLCZ 400 mg orally every d	AMB 40 mg orally every 4 h (6×/d)	68	48.71 (17-73)	2
Koh et al,^45^ 2002	Singapore	Hematopoietic stem cell transplant recipients (allogeneic and autologous HSCT)	FLCZ 200 mg orally every d	AMB 0.2 mg/kg IV every d	186	29.69 (4-63)	2
Lass-Florl et al,^46^ 2003	Austria	Patients with hematological malignant neoplasms receiving chemotherapy	ITCZ 5 mg/kg orally 2×/d	AMB 1000 mg orally 3×/d	106	43.94 (NA)	3
Laverdiere et al,^47^ 2000	Canada	Patients with hematological malignant neoplasms receiving chemotherapy or undergoing transplantation	FKCZ 400 mg orally every d	Placebo	266	46.31 (17-80)	2
Mahmoud et al,^48^ 2016	Egypt	Patients with acute leukemia receiving chemotherapy	MCFG 1 mg/kg IV every d	Placebo	70	7.35 (0-18)	2
Marks et al,^49^ 2011	International	Hematopoietic stem cell transplant recipients (allogeneic HSCT)	ITCZ 200 mg orally 2×/d	VOCZ 200 mg orally 2×/d	465	42.78 (11-70)	3
Marr et al,^50^ 2004	United States	Hematopoietic stem cell transplant recipients (allogeneic HSCT)	FLCZ 400 mg orally or IV every d	ITCZ 2.5 mg/kg orally 3×/d or 200 mg IV every d	299	NA	2
Mattiuzzi et al,^51^ 2003	United States	Patients with AML or MDS receiving chemotherapy	AMBL 3 mg/kg IV 3×/wk	FLCZ 200 mg orally 2×/d and ITCZ 200 mg/kg orally 2×/d	137	60.57 (19-84)	2
Mattiuzzi et al,^52^ 2006	United States	Patients with AML or MDS receiving chemotherapy	ITCZ 200 mg IV every d	CASP 50 mg IV every d	200	62.17 (17-82)	2
Mattiuzzi et al,^53^ 2011	United States	Patients with AML or MDS receiving chemotherapy	ITCZ 200 mg IV every d	VOCZ 300 mg IV 2×/d	123	59.42 (21-83)	2
Menichetti et al,^54^ 1999	Italy	Patients with hematological malignant neoplasms receiving chemotherapy or HSCT	ITCZ 200 mg orally 2×/d	Placebo	405	44.00 (17-79)	2
Morgenstern et al,^55^ 1999	United Kingdom	Patients with hematological malignanct neoplasms receiving chemotherapy or HSCT	FLCZ 100 mg orally every d	ITCZ 2.5 mg/kg orally 2×/d	445	44.55 (16-81)	2
Nucci et al,^56^ 2000	Brazil	Patients with hematological malignant neoplasms receiving chemotherapy or HSCT	ITCZ 100 mg orally 2×/d	Placebo	210	27.7 (5-67)	2
Oren et al,^57^ 2006	Israel	Patients with hematological diseases receiving chemotherapy or HSCT	FLCZ 400 mg orally or IV every d	ITCZ 200 mg orally or IV every d	195	49.49 (17-75)	3
Palmblad et al,^58^ 1992	Sweden	Patients with acute leukemia receiving chemotherapy	KTCZ 200 mg orally every d	Placebo	107	50.47 (15-74)	2
Park et al,^59^ 2016	Korea	Hematopoietic stem cell transplant recipients (allogeneic and autologous HSCT)	FLCZ 400 mg orally every d	MCFG 50 mg IV every d	250	46.66 (20-64)	2
Penack et al,^60^ 2006	Germany	Patients with hematological diseases receiving chemotherapy or HSCT	AMBL 50 mg IV every other d	Placebo	132	53.76 (21-77)	3
Perfect et al,^61^ 1992	United States	Bone marrow transplant recipients (autologous HSCT)	AMB 0.1 mg/kg IV every d	Placebo	182	38.75 (24-55)	2
Philpott-Howard et al,^62^ 1993	International	Patients with hematological diseases receiving chemotherapy or HSCT	FLCZ 50 mg orally 2×/d	Polyene, nystatin 1 mIU orally 4×/d or AMB 500 mg orally 4×/d	536	45.9 (11-87)	3
Rijnders et al,^63^ 2008	Netherlands	Patients with hematological diseases receiving chemotherapy or HSCT	AMBL 12.5 mg INH every d	Placebo	271	49.49 (18-74)	1
Riley et al,^64^ 1994	United States	Bone marrow transplant recipients (allogeneic and autologous HSCT)	AMB 0.1 mg/kg IV every d	Placebo	35	38.00 (10-52)	3
Rotstein et al,^65^ 1999	Canada	Patients with hematological malignant neoplasms receiving chemotherapy or undergoing HSCT	FLCZ 400 mg orally every d	Placebo	304	46.40 (17-80)	2
Sawada et al,^66^ 2009	Japan	Patients with hematological diseases receiving chemotherapy or HSCT	FlCZ 10 mg/kg IV every d	MCFG 2 mg/kg IV every d	107	6.01 (NA)	2
Schaffner and Schaffner,^67^ 1995	Swizerland	Patients with AML or NHL receiving chemotherapy or HSCT	FLCZ 400 mg orally every d	Placebo	151	39.5 (17-71)	2
Schwartz et al,^68^ 1999	Germany	Patients with hematological malignant neoplasmscies receiving chemotherapy or HSCT	AMB 10 mg INH 2×/d	Placebo	382	46.81 (16-81)	1
Shen et al,^69^ 2013	China	Patients with AML or MDS receiving chemotherapy	FLCZ 400 mg orally every d	POCZ 200 mg orally TID	234	40.00 (15-68)	3
Slavin et al,^70^ 1995	Australia	Bone marrow transplant recipients (allogeneic and autologous HSCT)	FLCZ 400 mg orally every d	Placebo	300	36.35 (13-65)	2
Tollemar et al,^71^ 1993	Sweden	Bone marrow transplant recipients (allogeneic and autologous HSCT)	AMBL 1 mg/kg IV every d	Placebo	53	58.3 (NA)	2
Ullmann et al,^72^ 2007	International	Patients with grade II-IV acute GVHD or chronic GVHD	FLCZ 200 mg orally 3×/d	POCZ 200 mg orally 3×/d	600	41.30 (13-72)	2
Van Burik et al,^73^ 2004	United States	Hematopoietic stem cell transplant recipients (allogeneic and autologous HSCT)	FLCZ 400 mg IV every d	MCFG 50 mg IV every d	882	42.52 (0-73)	2
Vehreschild et al,^74^ 2007	Germany	Patients with AML receiving chemotherapy	VOCZ 200 mg orally 2×/d	Placebo	25	53.60 (18-73)	3
Vreugdenhil et al,^75^ 1993	Netherlands	Patients with hematological malignant neoplasms receiving chemotherapy	ITCZ 200 mg orally 2×/d	Placebo	92	49.5 (15-75)	2
Wingard et al,^76^ 1987	United States	Patients with hematological diseases receiving chemotherapy or HSCT	MICZ 5 mg/kg IV every d	Placebo	208	33.93 (6-75)	2
Wingard et al,^77^ 2010	United States	Hematopoietic stem cell transplant recipients (allogeneic HSCT)	FLCZ 400 mg orally every d	VOCZ 200 mg orally 2×/d	600	43.00 (2-65)	3
Winston et al,^78^ 1993	United States	Patients with acute leukemia receiving chemotherapy	FLCZ 400 mg orally every d or 200 mg IV 2×/d	Placebo	256	43.47 (17-82)	2
Winston et al,^79^ 2003	United States	Hematopoietic stem cell transplant recipients (allogeneic HSCT)	FLCZ 400 mg orally or IV every d	ITCZ 200 mg IV every d	138	39.54 (14-63)	1
Wolff et al,^80^ 2000	United States	Bone marrow transplant recipients (allogeneic and autologous HSCT)	FLCZ 400 mg orally or IV every d	AMB 0.2 mg/kg IV every d	355	42.55 (18-68)	2
Yamac et al,^81^ 1995	Turkey	Patients with hematological diseases receiving chemotherapy	FLCZ 200 mg orally 2×/d	Placebo	70	49.41 (16-68)	2
Young et al,^82^ 1999	International	Patients with leukemia receiving chemotherapy	FLCZ 200 mg orally every d	Polyene, nystatin 8 mIU orally every d	164	43.23 (17-80)	2

Abbrevations: AMB, amphotericin B; AMBL, liposomal amphotericin B; CASP, caspofungin; FLCZ, fluconazole; KTCZ, ketoconazole; ITCZ, itraconazole; INH, inhalation; IV, intravenously; MCFG, micafungin; NA, not available; POCZ, posaconazole; RoB2, revised tool for risk of bias; VOCZ, voriconazole.

### Network Geometry and Synthesis of Results

**Figure 1.**
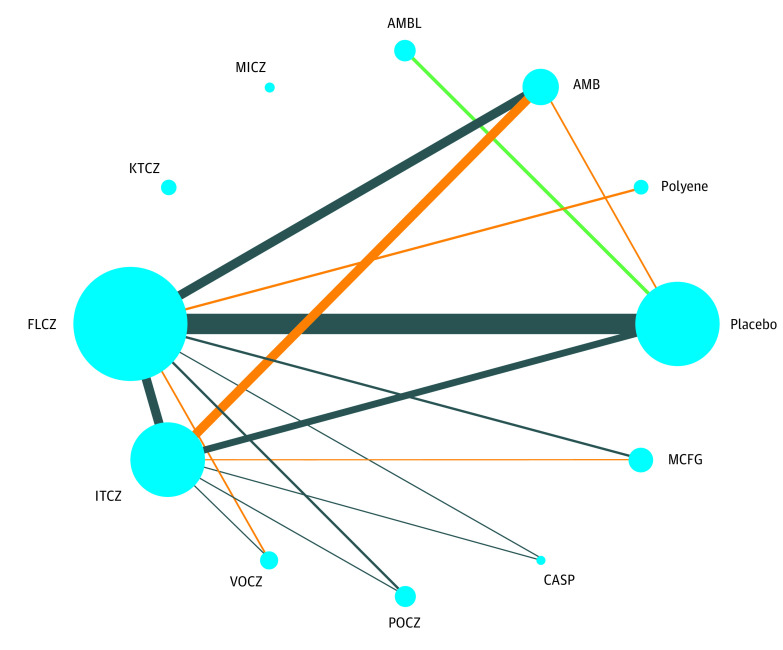
Schematic of the Network of Evidence Used in Network Meta-analysis for Invasive Fungal Infections AMB indicates conventional amphotericin B; AMBL, liposomal amphotericin B; KTCZ, ketoconazole; FLCZ, fluconazole; ITCZ, itraconazole; VOCZ, voriconazole; POCZ, posaconazole; CASP, caspofungin; and MCFG, micafungin.

The network geometry for each outcome is shown in eFigure 3 in the Supplement: fungal infections included 12 groups, 69 studies, and 14 789 patients; IFIs included 12 groups (Figure 1), 64 studies, and 12 943 patients; proven IFIs included 11 groups, 37 studies, and 7179 patients; invasive candidiasis included 12 groups, 45 studies, and 9838 patients; invasive aspergillosis included 12 groups, 40 studies, and 7958 patients; mortality included 12 groups, 69 studies, and 14 789 patients; fungi-related deaths included 12 groups, 45 studies, and 8636 patients; and withdrawal included 11 groups, 39 studies, and 9056 patients. Indirect and mixed-treatment comparisons are shown as forest plots (eFigure 4 in the Supplement).

**Figure 2.**
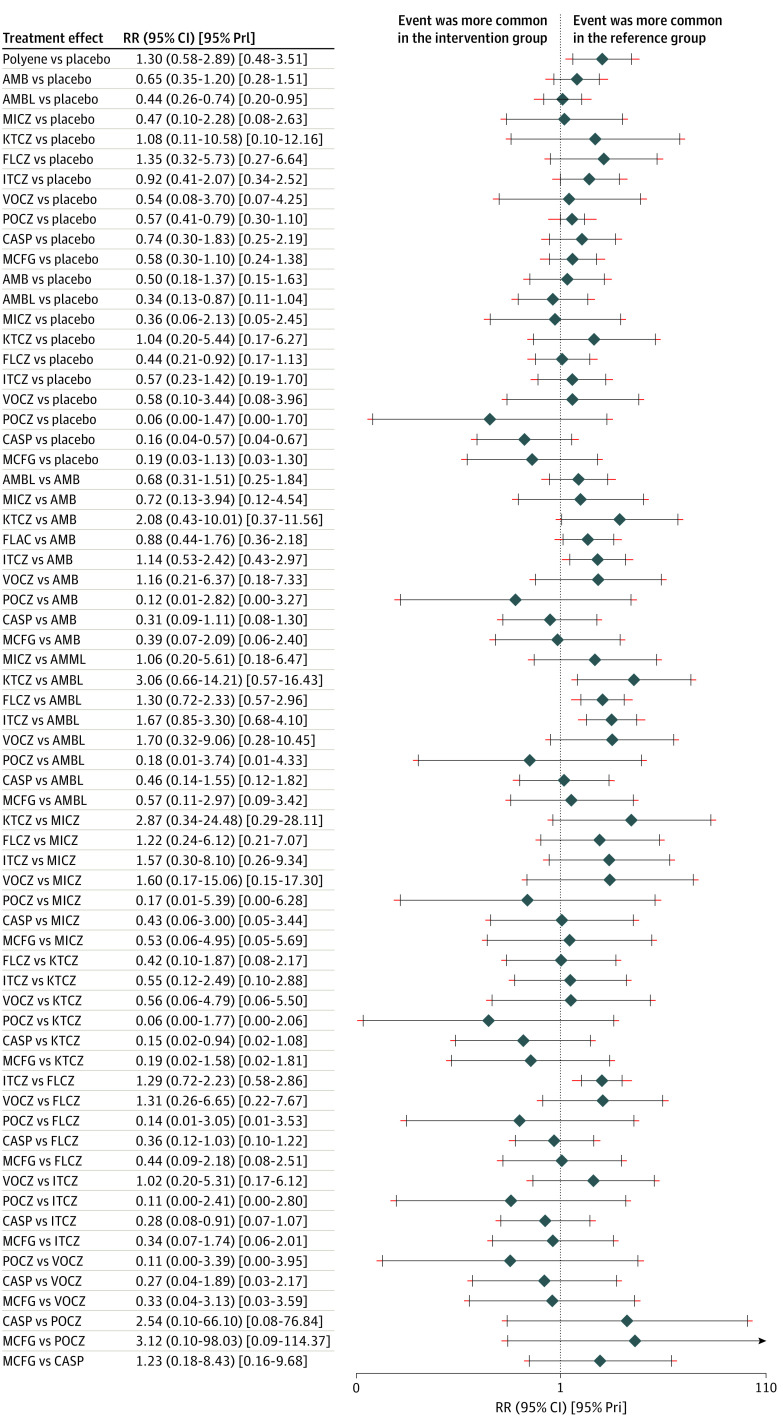
Forest Plot of Invasive Fungal Infections The dotted line indicates null effect; diamonds, relative risk (RR); and black whiskers, 95% CI; red whiskers, 95% predicted interval (PrI); AMB, conventional amphotericin B; AMBL, liposomal amphotericin B; KTCZ, ketoconazole; FLCZ, fluconazole; ITCZ, itraconazole; VOCZ, voriconazole; POCZ, posaconazole; CASP, caspofungin; and MCFG, micafungin.

The SUCRA value and rank of each agent for each outcome are shown in Table 2. Regarding IFIs, posaconazole was the approach with the highest ranking (SUCRA, 86.7%; mean rank, 2.5). The 2 approaches with the next-highest rankings were caspofungin (SUCRA, 84.2%) and micafungin (SUCRA, 76.4%). Posaconazole was associated with a significant reduction in IFIs (RR, 0.57; 95% CI, 0.42-0.79) and invasive aspergillosisus infections (RR, 0.36; 95% CI, 0.15-0.85) compared with placebo (Figure 2). Regarding mortality, the treatment ranked highest was micafungin (SUCRA, 90.0%; mean rank, 2.1). Voriconazole ranked second (SUCRA, 73.8%), and posaconazole ranked third (SUCRA, 68.5%).

**Table 2.** SUCRA Values and Mean Rank for All Outcomes

Measure	Fungal infections	IFIs	Proven IFIs	Invasive candidiasis	Invasive aspergillosis	Mortality	Fungi-related death	Withdrawal
**Overall**								
Placebo								
SUCRA, %	6.2	19.2	25.3	11.6	40.9	38.0	33.7	45.2
Mean rank	11.3	9.9	8.5	10.7	7.5	7.8	8.3	6.5
Polyene								
SUCRA, %	27.5	12.6	37.0	31.0	18.0	15.0	22.6	72.0
Mean rank	9.0	10.6	7.3	8.6	10.0	10.4	9.5	3.8
Amphotericin B								
SUCRA, %	37.5	41.0	14.1	3.6	69.3	42.7	28.3	50.5
Mean rank	7.9	7.5	9.6	11.6	4.4	7.3	8.9	5.9
Liposomal amphotericin B								
SUCRA, %	46.8	61.5	59.7	44.2	37.8	61.8	78.8	4.3
Mean rank	6.8	5.2	5.0	7.1	7.8	5.2	3.3	10.6
Miconazole								
SUCRA, %	76.6	55.2	NA	60.7	42.3	44.5	58.5	NA
Mean rank	3.6	5.9	NA	5.3	7.3	7.1	5.6	NA
Ketoconazole								
SUCRA, %	58.4	17.1	6.3	26.6	63.4	15.4	7.5	63.0
Mean rank	5.6	10.1	10.4	9.1	5.0	10.3	11.2	4.7
Fluconazole								
SUCRA, %	48.8	45.8	60.5	67.1	24.2	49.0	51.6	41.0
Mean rank	6.6	7.0	4.9	4.6	9.3	6.6	6.3	6.9
Itraconazole								
SUCRA, %	28.4	64.6	75.4	80.5	22.4	47.1	64.3	38.0
Mean rank	8.9	4.9	3.5	3.1	9.5	6.8	4.9	7.2
Voriconazole								
SUCRA, %	30.5	36.9	81.5^a^	67.1	51.2	73.8	75.0	78.1^a^
Mean rank	8.6	8.4	2.9	4.6	6.4	3.9	3.8	3.2
Posaconazole								
SUCRA, %	82.9	86.7^a^	78.6	62.6	87.8^a^	68.5	76.2^a^	17.5
Mean rank	2.9	2.5	3.1	5.1	2.3	4.5	3.6	9.2
Caspofungin								
SUCRA, %	84.9^a^	84.2	35.1	88.5^a^	78.6	54.2	36.9	67.6
Mean rank	2.7	2.7	7.5	2.3	3.4	6.0	7.9	4.2
Micafungin								
SUCRA, %	71.4	75.0	76.4	56.6	64.0	90.0^a^	66.6	72.7
Mean rank	4.1	3.7	3.4	5.8	5.0	2.1	4.7	3.7
**Transplantation**								
Placebo								
SUCRA, %	8.5	7.4	4.5	2.3	41.8	25.7	25.6	45.8
Mean rank	8.3	7.5	7.7	6.9	4.5	5.5	4.7	4.8
Polyene								
SUCRA, %	NA	NA	NA	NA	NA	NA	NA	NA
Mean rank	NA	NA	NA	NA	NA	NA	NA	NA
Amphotericin B								
SUCRA, %	44.1	48.2	44.5	75.9	22.6	94.2^a^	23.4	48.0
Mean rank	5.5	4.6	4.9	2.4	5.6	1.3	4.8	4.6
Liposomal amphotericin B								
SUCRA, %	14.3	25.2	40.7	30.7	40.7	36.3	32.7	6.4
Mean rank	7.9	6.2	5.1	5.2	4.6	4.8	4.4	7.6
Miconazole								
SUCRA, %	NA	NA	NA	NA	NA	NA	NA	NA
Mean rank	NA	NA	NA	NA	NA	NA	NA	NA
Ketoconazole								
SUCRA, %	42.8	NA	NA	NA	NA	NA	NA	NA
Mean rank	5.6	NA	NA	NA	NA	NA	NA	NA
Fluconazole								
SUCRA, %	48.3	44.2	44.2	56.8	37.8	44.5	59.0	62.3
Mean rank	5.1	4.9	4.9	3.6	4.7	4.3	3.0	3.6
Itraconazole								
SUCRA, %	69.7	66.9	59.5	77.8^a^	54.2	32.3	72.7	35.0
Mean rank	3.4	3.3	3.8	2.3	3.7	5.1	2.4	5.6
Voriconazole								
SUCRA, %	75.1^a^	68.4	71.6^a^	60.9	70.5	49.2	NA	89.4^a^
Mean rank	3.0	3.2	3.0	3.3	2.8	4.0	NA	1.7
Posaconazole								
SUCRA, %	73.0	76.3^a^	69.5	NA	NA	NA	NA	28.2
Mean rank	3.2	2.7	3.1	NA	NA	NA	NA	6.0
Caspofungin								
SUCRA, %	NA	NA	NA	NA	NA	NA	NA	NA
Mean rank	NA	NA	NA	NA	NA	NA	NA	NA
Micafungin								
SUCRA, %	74.2	63.4	65.5	45.6	82.3^a^	67.7	86.6^a^	84.9
Mean rank	3.1	3.6	3.4	4.3	2.1	2.9	1.7	2.1
**AML or MDS**								
Placebo								
SUCRA, %	73.5	74.0	NA	NA	NA	22.5	NA	NA
Mean rank	2.9	2.8	NA	NA	NA	6.4	NA	NA
Polyene								
SUCRA, %	NA	NA	NA	NA	NA	NA	NA	NA
Mean rank	NA	NA	NA	NA	NA	NA	NA	NA
Amphotericin B								
SUCRA, %	11.9	14.3	54.2^a^	45.8	43.5	66.7	55.9	NA
Mean rank	7.2	7.0	2.4	3.7	3.8	3.3	3.6	NA
Liposomal amphotericin B								
SUCRA, %	18.0	11.5	NA	21.0	55.4	44.4	59.4	44.6
Mean rank	6.7	7.2	NA	4.9	3.2	4.9	3.4	3.8
Miconazole								
SUCRA, %	NA	NA	NA	NA	NA	NA	NA	NA
Mean rank	NA	NA	NA	NA	NA	NA	NA	NA
Ketoconazole								
SUCRA, %	NA	NA	NA	NA	NA	NA	NA	NA
Mean rank	NA	NA	NA	NA	NA	NA	NA	NA
Fluconazole								
SUCRA, %	52.0	53.1	49.0	50.0	26.5	56.1	59.8	71.9
Mean rank	4.4	4.3	2.5	3.5	4.7	4.1	3.4	2.4
Itraconazole								
SUCRA, %	42.4	43.5	53.4	55.7	14.6	28.5	18.2	28.5
Mean rank	5.0	5.0	2.4	3.2	5.3	6.0	5.9	4.6
Voriconazole								
SUCRA, %	82.7	83.1	NA	NA	NA	55.8	58.5	6.8
Mean rank	2.2	2.2	NA	NA	NA	4.1	3.5	5.7
Posaconazole								
SUCRA, %	83.4^a^	83.3^a^	NA	46.4	84.0^a^	80.1^a^	83.0^a^	89.5^a^
Mean rank	2.2	2.2	NA	3.7	1.8	2.4	2.0	1.5
Caspofungin								
SUCRA, %	35.9	37.2	43.4	81.1^a^	76.0	45.8	15.4	58.7
Mean rank	5.5	5.4	2.7	1.9	2.2	11.0	6.1	3.1
Micafungin								
SUCRA, %	NA	NA	NA	NA	NA	NA	NA	NA
Mean rank	NA	NA	NA	NA	NA	NA	NA	NA
**Allo-HSCT**								
Placebo								
SUCRA, %	NA	NA	NA	NA	NA	NA	NA	NA
Mean rank	NA	NA	NA	NA	NA	NA	NA	NA
Polyene								
SUCRA, %	NA	NA	NA	NA	NA	NA	NA	NA
Mean rank	NA	NA	NA	NA	NA	NA	NA	NA
Amphotericin B								
SUCRA, %	NA	NA	NA	NA	NA	NA	NA	NA
Mean rank	NA	NA	NA	NA	NA	NA	NA	NA
Liposomal amphotericin B								
SUCRA, %	NA	NA	NA	NA	NA	NA	NA	NA
Mean rank	NA	NA	NA	NA	NA	NA	NA	NA
Miconazole								
SUCRA, %	NA	NA	NA	NA	NA	NA	NA	NA
Mean rank	NA	NA	NA	NA	NA	NA	NA	NA
Ketoconazole								
SUCRA, %	NA	NA	NA	NA	NA	NA	NA	NA
Mean rank	NA	NA	NA	NA	NA	NA	NA	NA
Fluconazole								
SUCRA, %	4.4	4.7	10.3	27.9	15.9	60.1	22.4	50.8
Mean rank	2.9	2.9	2.8	2.4	2.7	1.8	1.8	2.0
Itraconazole								
SUCRA, %	65.3	66.0	64.6	85.8^a^	40.9	14.5	77.6^a^	0.0
Mean rank	1.7	1.7	1.7	1.3	2.2	2.7	1.2	3.0
Voriconazole								
SUCRA, %	80.3^a^	79.3^a^	75.1^a^	36.3	93.3^a^	75.4^a^	NA	99.2^a^
Mean rank	1.4	1.4	1.5	2.3	1.1	1.5	NA	1.0
Posaconazole								
SUCRA, %	NA	NA	NA	NA	NA	NA	NA	NA
Mean rank	NA	NA	NA	NA	NA	NA	NA	NA
Caspofungin								
SUCRA, %	NA	NA	NA	NA	NA	NA	NA	NA
Mean rank	NA	NA	NA	NA	NA	NA	NA	NA
Micafungin								
SUCRA, %	NA	NA	NA	NA	NA	NA	NA	NA
Mean rank	NA	NA	NA	NA	NA	NA	NA	NA

Abbreviations: AML, acute myeloid leukemia; HSCT, hematopoietic stem cell transplantation; MDS, myelodysplastic syndrome; NA, not available; SUCRA, surface under the cumulative ranking curve.

^a^ Ranking first among agents.

Caspofungin (SUCRA, 84.9%) treatment ranked the highest for reducing fungal infections. Posaconazole ranked highest in preventing invasive aspergillosis (SUCRA, 87.8%). Caspofungin was ranked highest for preventing invasive candidiasis (SUCRA, 88.5%), and liposomal amphotericin B ranked the highest for reducing fungi-related deaths (SUCRA, 78.8%). Voriconazole was associated with a significant reduction in invasive candidiasis (RR, 0.15; 95% CI, 0.09-0.26) compared with placebo (eFigure 5 in the Supplement). Voriconazole was ranked highest for having the lowest incidence of withdrawal (SUCRA, 78.1%). Posaconazole was associated with a higher incidence of withdrawal because of the adverse effects of the drug (SUCRA, 17.5%; mean rank, 9.2) (eFigure 6 in the Supplement).

### Subgroup Analyses

Because extensive categories of patients were included, we evaluated whether the prophylactic outcomes and tolerance of agents varied in different patient populations (patients with acute myeloid leukemia [AML] or myelodysplastic syndrome [MDS] or undergoing HSCT or allo-HSCT). Considering efficacy and tolerance, voriconazole was ranked as the best choice for patients undergoing HSCT; this result was also found in the allo-HSCT population. However, posaconazole was ranked as the best choice for patients with AML or MDS.

### Heterogeneity, Inconsistency, and Small-Study Effects

Heterogeneity and inconsistency are shown in Table 3. Heterogeneity was low for IFIs and mortality. In contrast, heterogeneity was reasonable for the other outcomes (τ^2^ values from 0.1 to 0.4). Loop inconsistency for placebo, amphotericin B, and fluconazole was found for invasive candidiasis (indirect effect estimate, 2.53; 95% CI, 1.07-3.98; *P* = .001) (eFigure 7 in the Supplement). We used a funnel plot to visually demonstrate small-study effects (eFigure 8 in the Supplement).

**Table 3.** Tests for Inconsistency, Heterogeneity, and Small-Study Effects

Outcome	Inconsistency at the overall level		Heterogeneity (τ^2^)	Begg test *P* value
	χ^2^	*P* value		
Fungal infections	13.97	.45	0.118	.06
IFIs	10.04	.75	0.069	.40
Proven IFIs	8.77	.11	0.112	.40
Invasive candidiasis	13.51	.09	0.284	.35
Invasive aspergillosis	8.60	.48	0.192	.91
Mortality	6.20	.91	0.016	.48
Fungi-related death	13.05	.16	0.303	.93
Withdrawal	13.67	.06	0.334	.51

Abbreviation: IFI, invasive fungal infection.

## Discussion

In this systematic review and network meta-analysis, we combined direct and indirect evidence to compare antifungal prophylaxis options for patients with hematological disease or undergoing HSCT. Our analysis may provide some important information for clinical decision-making for antifungal prophylaxis in these patients. We derived 2 principal findings from our analysis: voriconazole may be the best choice for patients undergoing HSCT, and posaconazole may be the best prophylactic option for patients with AML or MDS. Posaconazole is recommended for IFIs during remission induction chemotherapy for AML and MDS, according to the Guidelines from the Infectious Diseases Working Party of the German Society for Haematology and Medical Oncology.^83^ Overall, posaconazole and voriconazole are recommended as the most reasonable options for the prevention of IFIs. The difference between agents may be meaningful and is not available from single trials, to our knowledge. For instance, voriconazole has not been directly compared with other drugs except for placebo, fluconazole, and itraconazole; however, this network meta-analysis compared voriconazole, as well as posaconazole, with other drugs indirectly.

Posaconazole is recommended for the prevention of IFIs regardless of tolerance. The most commonly reported treatment-related adverse effects of oral posaconazole were digestive tract symptoms, including nausea, vomiting, and gastrointestinal upset.^84^ The most common cause for discontinuation was severe nausea or gastrointestinal upset.^85^ We noticed that the incidence of withdrawal was different in patients with AML or MDS and patients undergoing HSCT. We assumed that in patients undergoing HSCT, especially allo-HSCT, a high-dose pretreatment scheme, the use of cyclosporin and the incidence of gastrointestinal acute graft-vs-host disease (GVHD) would decrease the tolerance of posaconazole. The rate of adverse events that led to the discontinuation of posaconazole was 40% in the study by Chatter et al.^22^ In the posaconazole group, the rate of diarrhea was 67%, of nausea was 67%, and of vomiting was 29%. The rate of gastrointestinal adverse events was similar between the liposomal amphotericin B and posaconazole groups. A new route of administration through injection may be an option for patients who are unable to swallow or are intolerant of oral posaconazole.

Caspofungin is recommended to prevent invasive candidiasis, and the same results were confirmed in patients with AML or MDS. There were no relevant data from patients undergoing HSCT. An echinocandin drug is recommended as the initial therapy for candidemia, according to the clinical practice guidelines for the management of candidiasis from the Infectious Diseases Society of America.^86^ In our analysis, posaconazole was ranked the best choice for preventing invasive *Aspergillus* infections, followed by caspofungin. According to the guidelines for the diagnosis and management of aspergillosis from the Infectious Diseases Society of America, posaconazole, voriconazole, and micafungin are recommended for invasive aspergillosis prevention.^87^ However, in our analysis, it appeared that caspofungin treatment was associated with a better outcome than micafungin and voriconazole in preventing aspergillosis. Concerning the prevention of fungi-related death, treatment with liposomal amphotericin B might be associated with a better outcome, followed by posaconazole and voriconazole. There was no significant difference in fungi-related death with posaconazole.

Some previous studies have summarized the data from the literature on antifungal prophylaxis in hematological disease.^88,89,90^ These network meta-analyses did not take into account antifungal prophylaxis in other high-risk groups, such as patients with GVHD.^91^ With regard to GVHD, the risk of IFIs appears particularly prominent in patients with high-grade acute GVHD or steroid-dependent chronic GVHD.^91^ Our network meta-analysis has taken into account antifungal prophylaxis in patients with GVHD. A high number of patients with solid tumor without HSCT therapy were included in previous network meta-analyses. In the study by Ninane et al,^92^ solid tumors were present in more than 20% of patients. We did not consider this study appropriate for a network meta-analysis for antifungal prophylaxis, as routine antifungal prophylaxis is not recommended in patients with solid tumors.^91^ The evidence shown by Leonart et al^88^ focused on double-blind trials, and the study by Zhao et al^89^ focused on triazole agents. Therefore, the conclusions of the comparisons in those reports cannot be compared with the results in this meta-analysis.

Inconsistency refers to the differences between direct and various indirect effect estimates for the same comparison. Stata tests for inconsistency have 2 levels^12^: overall inconsistency, in which the level of inconsistency is computed according to the type of between-treatment comparison for all cases and a local approach, in which each treatment is individually examined. Local inconsistency because of loop inconsistency in placebo, amphotericin B, and fluconazole was found for invasive candidiasis in our analysis. There are 4 causes of inconsistency: chance, bias in head-to-head comparisons, bias in indirect comparisons, and genuine diversity.^93^ According to Higgins et al^11^ loop inconsistency refers to a difference between direct and indirect comparisons. The study by Behre et al^16^ may be the source of the loop inconsistency because aerosol amphotericin B inhalation treatment was used. When this study was excluded from the analysis, the difference between direct and indirect comparisons of the treatments for invasive candidiasis was not significant.

The interpretations of the results were based on SUCRA values and ranking in our study. Although rankings are appealing, they may be incorrectly emphasize particular treatments as being clinically useful. The uncertainty present in the rankings may be neglected in considering the best treatment, and the rankings may give a false sense that some interventions are superior to others. A PRISMA-NMA statement has suggested that more attention should be paid to the relative effect estimates, rather than the rankings, because a good rank does not necessarily translate to a clinically relevant effect.^6^ Although the usefulness of rankings is currently debated, rankings will probably continue to be reported. Reporting all probabilities for each intervention with each possible rank is one way to convey the uncertainty in the rank ordering.^94^ The treatment effects and rankings also depend on the number of treatments and trials in the network.^95^

### Limitations

Our study has some limitations. First, an improved understanding of antifungal pharmacology, pharmacokinetics, and pharmacodynamics has resulted in therapeutic drug monitoring becoming a valuable adjunct to the administration of some antifungal agents. We could not perform an analysis of therapeutic drug monitoring to evaluate the efficacy and adverse effects of antifungal prophylaxis or justify why therapeutic drug monitoring should not be performed with antifungal prophylaxis if it is strongly recommended with antifungal treatment because of the limited data. Second, the follow-up time of most studies was too short to determine the survival benefits from antifungal prophylaxis. Third, a limited number of head-to-head trials have investigated posaconazole and voriconazole. Fourth, the characteristics of patients and treatments were heterogeneous among the various randomized clinical trials. Although our subgroup analyses found different results among different patient populations, the data did not allow us to perform more detailed analyses, such as those for different ages and races/ethnicities. We also could not perform a more stratified analysis taking into consideration the dosage form and dose of agents. Therefore, the evidence derived from this meta-analysis should be used with caution for shared decision-making. However, our study provides important data from which future practice-changing prospective trials can be designed.

## Conclusions

This network meta-analysis assessed the performance of various antifungal prophylaxis treatment in patients with hematological disease or undergoing HSCT. Our findings suggest that, in terms of the prevention of IFIs and tolerance, voriconazole may be the best prophylactic option for patients undergoing HSCT, and posaconazole may be the best prophylactic option for patients with AML or MDS.

## Notes

Supplement. **eAppendix.** Search Strategy **eFigure 1.** PRISMA Flowchart **eFigure 2.** Quality Assessment **eFigure 3.** Network Geometry of All Outcomes **eFigure 4.** Forest Plots of All Outcomes **eFigure 5.** League Tables of All Outcomes **eFigure 6.** Cluster Rank Combined the SUCRA **eFigure 7.** Inconsistency Plot of All Outcomes **eFigure 8.** Funnel Plots of All Outcomes
