# Supplementary material for: Comparison of Antifungal Prophylaxis Drugs in Patients With Hematological Disease or Undergoing Hematopoietic Stem Cell Transplantation: A Systematic Review and Network Meta-analysis
Source: JAMA Netw Open. 2020 Oct 8;3(10):e2017652. doi: 10.1001/jamanetworkopen.2020.17652 (PMC7545296; doi:10.1001/jamanetworkopen.2020.17652)
Supplement: Supplement. — eAppendix. Search Strategy eFigure 1. PRISMA Flowchart eFigure 2. Quality Assessment eFigure 3. Network Geometry of All Outcomes eFigure 4. Forest Plots of All Outcomes eFigure 5. League Tables of All Outcomes eFigure 6. Cluster Rank Combined the SUCRA eFigure 7. Inconsistency Plot of All Outcomes eFigure 8. Funnel Plots of All Outcomes [file jamanetwopen-e2017652-s001.pdf]

Supplemental Online Content

Wang J, Zhou M, Xu JY, Zhou RF, Chen B, Wan Y. Comparison of antifungal prophylaxis drugs in patients with hematological disease or undergoing hematopoietic stem cell transplantation: a systematic review and network meta-analysis. *JAMA Netw Open*. 2020;3(10):e2017652. doi:10.1001/jamanetworkopen.2020.17652

eAppendix. Search Strategy
eFigure 1. PRISMA Flowchart
eFigure 2. Quality Assessment
eFigure 3. Network Geometry of All Outcomes
eFigure 4. Forest Plots of All Outcomes
eFigure 5. League Tables of All Outcomes
eFigure 6. Cluster Rank Combined the SUCRA
eFigure 7. Inconsistency Plot of All Outcomes
eFigure 8. Funnel Plots of All Outcomes

This supplemental material has been provided by the authors to give readers additional information about their work.

© 2020 Wang J et al. *JAMA Netw Open.*

eAppendix. Search Strategies

#1 ((clinical AND trial)[Title/Abstract] OR clinical trial[Title/Abstract] OR random*[Title/Abstract] OR random allocation[Title/Abstract])

#2 (“fungal infection*”[Title/Abstract] OR aspergillosis[Title/Abstract] OR blastomycosis[Title/Abstract] OR candidiasis[Title/Abstract] OR andidosis[Title/Abstract] OR coccidioidomycosis[Title/Abstract] OR cryptococcosis[Title/Abstract] OR histoplasmosis[Title/Abstract] OR mucormycosis[Title/Abstract] OR zygomycosis[Title/Abstract] OR paracoccidioidomycosis[Title/Abstract] OR “penicillium marneffei”[Title/Abstract] OR sporotrichosis[Title/Abstract] OR hyalohyphomycosis[Title/Abstract] OR fusariosis[Title/Abstract] OR fusarium[Title/Abstract] OR scedosporium[Title/Abstract] OR monosporium[Title/Abstract] OR phaeohyphomycosis[Title/Abstract] OR alternaria[Title/Abstract] OR bipolaris[Title/Abstract] OR curvularia[Title/Abstract] OR exserohilum[Title/Abstract] OR cladophialophora[Title/Abstract] OR xylohypha[Title/Abstract] OR trichosporonosis[Title/Abstract] OR malassezia[Title/Abstract] OR pityrosporum[Title/Abstract] OR pneumocystis[Title/Abstract] OR pneumocystosis[Title/Abstract] OR pseudallescherias[Title/Abstract] OR allescherias[Title/Abstract] OR petriellidium[Title/Abstract])

#3 (antifungal[Title/Abstract] OR “antifungal agent*” OR amphotericin*[Title/Abstract] OR albaconazol*[Title/Abstract] OR UR9825[Title/Abstract] OR UR 9825[Title/Abstract] OR UR-9825[Title/Abstract] OR ketoconazol*[Title/Abstract] OR R41400[Title/Abstract] OR R 41400[Title/Abstract] OR R-41400[Title/Abstract] OR fluconazol*[Title/Abstract] OR UK49858[Title/Abstract] OR UK 49858[Title/Abstract] OR UK-49858[Title/Abstract] OR isavuconazol*[Title/Abstract] OR BAL8557[Title/Abstract] OR BAL 8557[Title/Abstract] OR BAL-8557[Title/Abstract] OR itraconazol*[Title/Abstract] OR R51211[Title/Abstract] OR R 51211[Title/Abstract] OR R-51211[Title/Abstract] OR posaconazol*[Title/Abstract] OR SCH56592[Title/Abstract] OR SCH 56592[Title/Abstract] OR SCH-56592[Title/Abstract] OR ravuconazol*[Title/Abstract] OR BMS207147[Title/Abstract] OR BMS 207147[Title/Abstract] OR BMS-207147[Title/Abstract] OR ER30346[Title/Abstract] OR ER 30346[Title/Abstract] OR ER-30346[Title/Abstract]
OR voriconazol*[Title/Abstract] OR UK109496[Title/Abstract] OR UK 109496[Title/Abstract] OR UK-109496[Title/Abstract] OR aminocandin*[Title/Abstract] OR HMR3270[Title/Abstract] OR HMR 3270[Title/Abstract] OR HMR-3270[Title/Abstract] OR IP960[Title/Abstract] OR IP-960[Title/Abstract] OR IP 960[Title/Abstract] OR anidulafungin*[Title/Abstract] OR LY303366[Title/Abstract] OR LY 303366[Title/Abstract] OR LY-303366[Title/Abstract] OR caspofungin*[Title/Abstract] OR MK0991[Title/Abstract] OR MK 0991[Title/Abstract] OR MK-0991[Title/Abstract] OR L743872[Title/Abstract] OR L 743872[Title/Abstract] OR L-743872[Title/Abstract] OR micafungin*[Title/Abstract] OR FK463[Title/Abstract] OR FK 463[Title/Abstract] OR FK-463[Title/Abstract] OR flucytosin*[Title/Abstract] OR nystatin*[Title/Abstract]))

© 2020 Wang J et al. *JAMA Netw Open.*

eFigure 1. PRISMA Flowchart

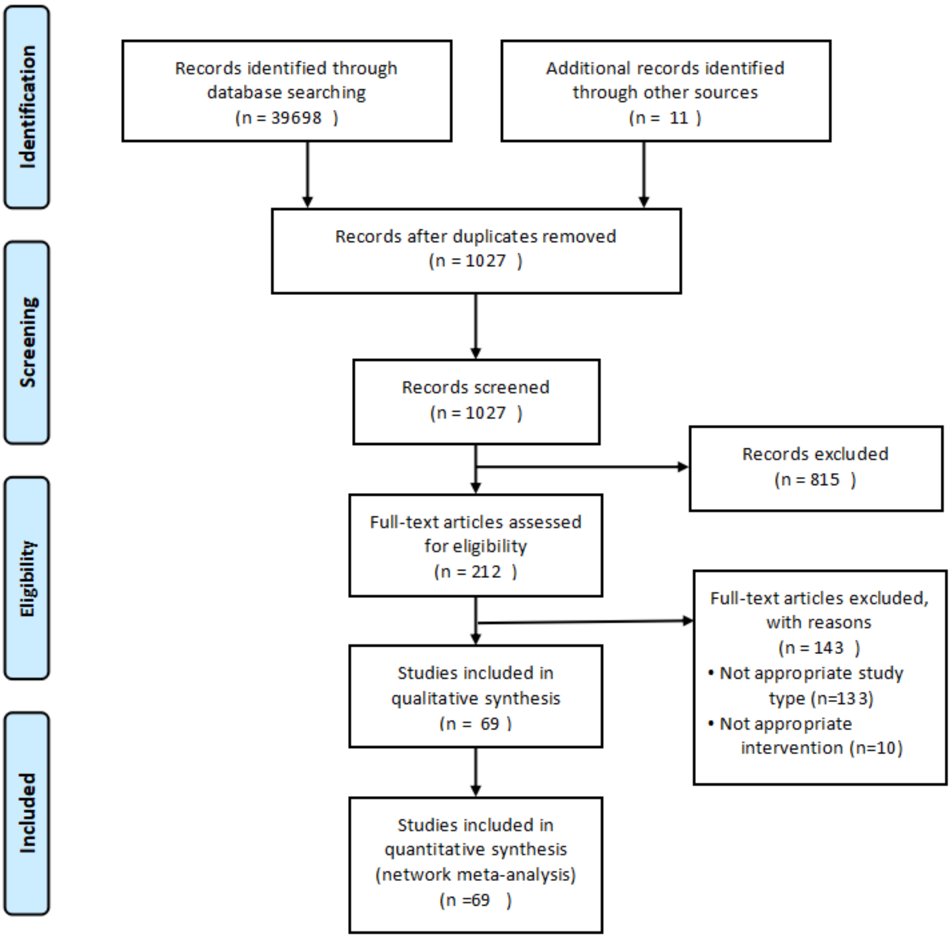

© 2020 Wang J et al. *JAMA Netw Open.*

eFigure 2. Quality Assessment

Study	Randomisation process	Deviations from intended interventions	Missing outcome data	Measurement of the outcome	Selection of the reported result	Overall bias
1 Akiyama, 1993	Low risk	Some concerns	Low risk	Low risk	Some concerns	Some concerns
2 Annaloro, 1995	Some concerns	Some concerns	Low risk	Low risk	Some concerns	Some concerns
3 Benhamou, 1991	Low risk	Some concerns	Low risk	Low risk	Some concerns	Some concerns
4 Bodey, 1994	Some concerns	Low risk	Low risk	Low risk	Some concerns	Some concerns
5 Boogaerts, 2001	Some concerns	Low risk	Low risk	Low risk	Low risk	Some concerns
6 Brincker, 1978	Some concerns	Some concerns	Low risk	Low risk	Some concerns	Some concerns
7 Brincker 1983	Some concerns	Some concerns	Low risk	Low risk	Low risk	Some concerns
8 Chaftari, 2012	Low risk	Some concerns	Low risk	Low risk	Low risk	Some concerns
9 Chandrasekar, 1994	Some concerns	Some concerns	Low risk	Low risk	High risk	High risk
10 Choi, 2005	High risk	Some concerns	Low risk	Low risk	Low risk	High risk
11 Cornely, 2007	Some concerns	Low risk	Low risk	Low risk	Low risk	Some concerns
12 Donnelly, 1984	Some concerns	Some concerns	Low risk	Low risk	Low risk	Some concerns
13 Egger, 1995	Low risk	Some concerns	Low risk	Low risk	High risk	High risk
14 Ellis, 1994	Some concerns	Some concerns	Low risk	Low risk	Low risk	Some concerns
15 Epstein, 2018	Some concerns	Some concerns	Low risk	Low risk	Low risk	Some concerns
16 Estey, 1984	High risk	Some concerns	Low risk	Low risk	Low risk	High risk
17 Fisher, 2019	Low risk	Some concerns	Low risk	Low risk	Low risk	Some concerns
18 Glasmacher, 2006	Low risk	Low risk	Low risk	Low risk	Low risk	Low risk
19 Goodman, 1992	Low risk	Some concerns	Low risk	Low risk	Low risk	Some concerns
20 Hansen, 1987	Some concerns	Some concerns	Low risk	Low risk	Low risk	Some concerns
21 Harousseau, 2000	Low risk	Low risk	Low risk	Low risk	Low risk	Low risk
22 Hayashi, 2014	Some concerns	Some concerns	Low risk	Low risk	Some concerns	Some concerns
23 Hiemenz, 2005	Some concerns	Some concerns	Low risk	Low risk	High risk	High risk
24 Hiramatsu, 2008	Some concerns	Low risk	Low risk	Low risk	Low risk	Some concerns
25 Huang, 2012	Low risk	Some concerns	Low risk	Low risk	High risk	High risk
26 Huijgens, 1999	Some concerns	Low risk	Low risk	Low risk	Low risk	Some concerns
27 Ito, 2007	Low risk	Some concerns	Low risk	Low risk	High risk	High risk
28 Kaptan, 2003	Some concerns	Some concerns	Low risk	Low risk	High risk	High risk
29 Kelsey, 1999	Low risk	Low risk	Low risk	Low risk	Low risk	Low risk
30 Kern, 1998	Some concerns	Some concerns	Low risk	Low risk	Low risk	Some concerns
31 Koh, 2002	Some concerns	Low risk	Low risk	Low risk	Low risk	Some concerns
32 Lass-Florl, 2003	Some concerns	Some concerns	Low risk	Low risk	High risk	High risk
33 Mahmoud, 2016	Some concerns	Some concerns	Low risk	Low risk	Some concerns	Some concerns
34 Marks, 2011	Low risk	Low risk	Low risk	Low risk	High risk	High risk
35 Marr, 2004	Some concerns	Low risk	Low risk	Low risk	Low risk	Some concerns
36 Mattiuzzi, 2003	Low risk	Some concerns	Low risk	Low risk	Low risk	Some concerns
37 Mattiuzzi, 2006	Some concerns	Low risk	Low risk	Low risk	Low risk	Some concerns
38 Mattiuzzi, 2011	Some concerns	Low risk	Low risk	Low risk	Low risk	Some concerns
39 Menichetti, 1999	Some concerns	Low risk	Low risk	Low risk	Low risk	Some concerns
40 Morgenstern, 1999	Low risk	Some concerns	Low risk	Low risk	Low risk	Some concerns
41 Nucci, 2000	Low risk	Some concerns	Low risk	Low risk	Low risk	Some concerns
42 Oren 2006	Some concerns	Some concerns	Low risk	Low risk	High risk	High risk
43 Palmblad, 1992	Some concerns	Some concerns	Low risk	Low risk	Low risk	Some concerns
44 Park, 2016	Some concerns	Some concerns	Low risk	Low risk	Low risk	Some concerns
45 Penack, 2006	Some concerns	High risk	Low risk	High risk	Low risk	High risk
46 Perfect, 1992	Some concerns	Some concerns	Low risk	Low risk	Low risk	Some concerns
47 Philpott-Howard, 1993	Some concerns	Low risk	Low risk	Low risk	High risk	High risk
48 Rijnders, 2008	Low risk	Low risk	Low risk	Low risk	Low risk	Low risk
49 Riley, 1994	Some concerns	Some concerns	Low risk	Low risk	High risk	High risk
50 Sawada, 2009	Some concerns	Some concerns	Low risk	Some concerns	Some concerns	Some concerns
51 Schaffner, 1995	Some concerns	Low risk	Low risk	Low risk	Low risk	Some concerns
52 Schwartz, 1999	Low risk	Low risk	Low risk	Low risk	Low risk	Low risk
53 Shen, 2013	Some concerns	Some concerns	Low risk	Low risk	High risk	High risk
54 Slavin, 1995	Some concerns	Low risk	Low risk	Low risk	Low risk	Some concerns
55 Tollemar, 1993	Some concerns	Some concerns	Low risk	Some concerns	Low risk	Some concerns
56 Ullmann, 2007	Some concerns	Low risk	Low risk	Low risk	Low risk	Some concerns
57 Van Burik, 2004	Some concerns	Low risk	Low risk	Low risk	Low risk	Some concerns
58 Vehreschild, 2007	Low risk	Some concerns	Low risk	Low risk	High risk	High risk
59 Vreugdenhil, 1993	Some concerns	Some concerns	Low risk	Low risk	Some concerns	Some concerns
60 Wingard, 1987	Some concerns	Some concerns	Low risk	Some concerns	Low risk	Some concerns
61 Wingard, 2010	Some concerns	Low risk	Low risk	Low risk	High risk	High risk
62 Winston, 1993	Some concerns	Low risk	Low risk	Low risk	Low risk	Some concerns
63 Winston, 2003	Low risk	Low risk	Low risk	Low risk	Low risk	Low risk
64 Wolff, 2000	Some concerns	Some concerns	Low risk	Low risk	Low risk	Some concerns
65 Yamac, 1995	Some concerns	Some concerns	Low risk	Some concerns	Low risk	Some concerns
66 Young, 1999	Some concerns	Some concerns	Low risk	Low risk	Low risk	Some concerns
67 Behre, 1995	Some concerns	Low risk	Low risk	Low risk	Low risk	Some concerns
68 Laverdiere, 2000	Some concerns	Some concerns	Low risk	Low risk	Low risk	Some concerns
69 Rotstein, 1999	Some concerns	Low risk	High risk	Low risk	Low risk	High risk

- \+ Low risk
- ? Some concerns
- ? High risk

© 2020 Wang J et al. *JAMA Netw Open.*

eFigure 3. Network Geometry of All Outcomes

Panel A. Fungal Infections

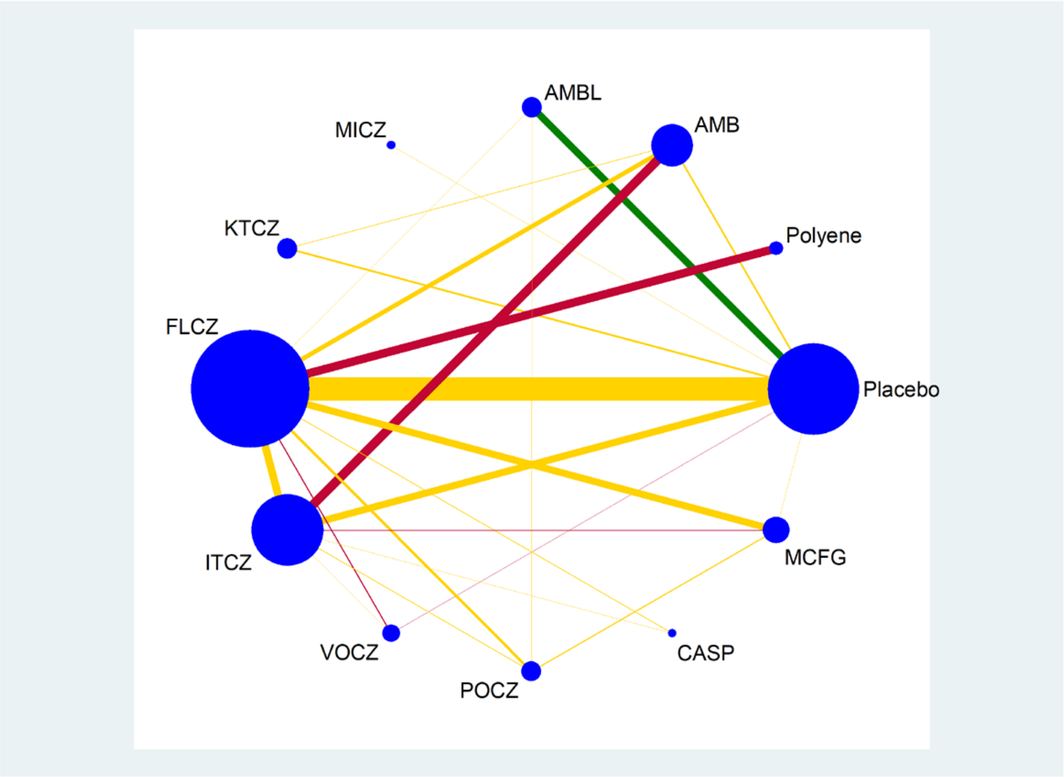

Legend: the blue nodes represent the interventions compared while the edges represent the direct comparisons available between pairs of interventions. The node sizes are weighted based on the number of patients in each arm while the edges are weighted using inverse variance. The edge colors (green or red) are weighted based on the quality of the studies included. A green edge indicates the comparison with high quality studies while a red line indicates comparison with low quality studies. AMB: conventional amphotericin B; AMBL: liposomal amphotericin B; MICZ: miconazole; KTCZ: ketoconazole; FLCZ: fluconazole; ITCZ: itraconazole; VOCZ: voriconazole; POCZ: posaconazole; CASP: caspofungin; MCFG: micafungin.

© 2020 Wang J et al. *JAMA Netw Open.*

Panel B. IFI

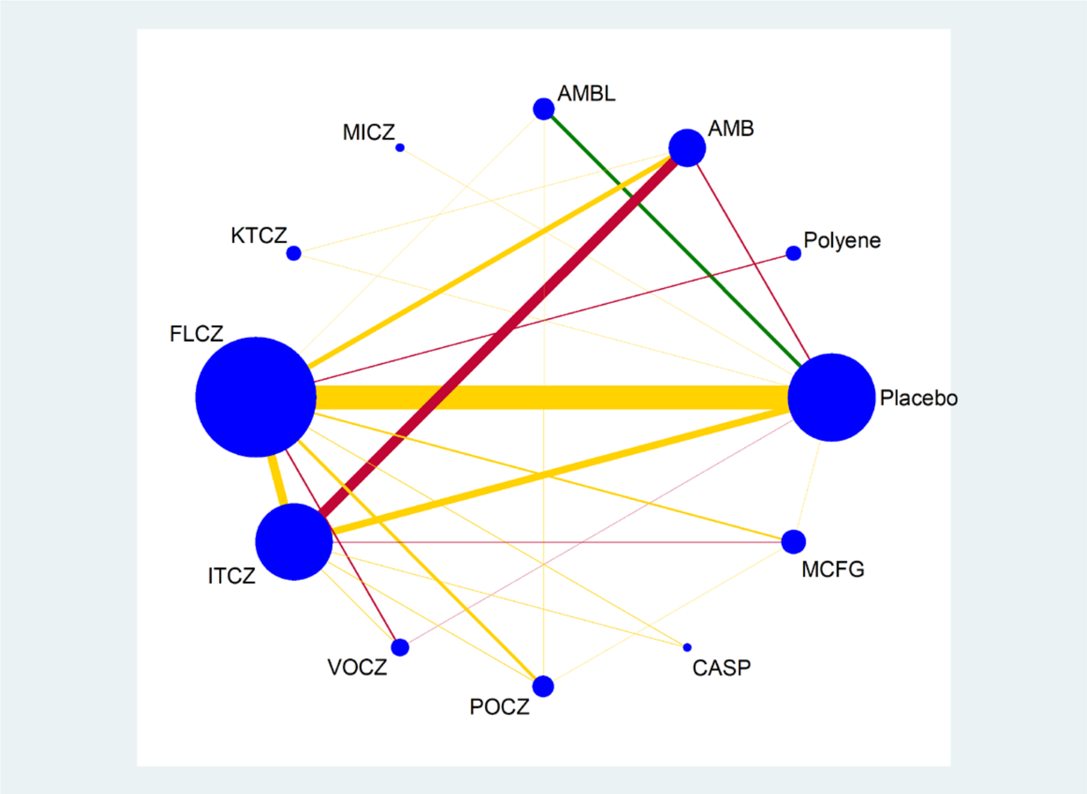

Legend: the blue nodes represent the interventions compared while the edges represent the direct comparisons available between pairs of interventions. The node sizes are weighted based on the number of patients in each arm while the edges are weighted using inverse variance. The edge colors (green or red) are weighted based on the quality of the studies included. A green edge indicates the comparison with high quality studies while a red line indicates comparison with low quality studies. AMB: conventional amphotericin B; AMBL: liposomal amphotericin B; MICZ: miconazole; KTCZ: ketoconazole; FLCZ: fluconazole; ITCZ: itraconazole; VOCZ: voriconazole; POCZ: posaconazole; CASP: caspofungin; MCFG: micafungin.

© 2020 Wang J et al. *JAMA Netw Open.*

Panel C. Proven IFI

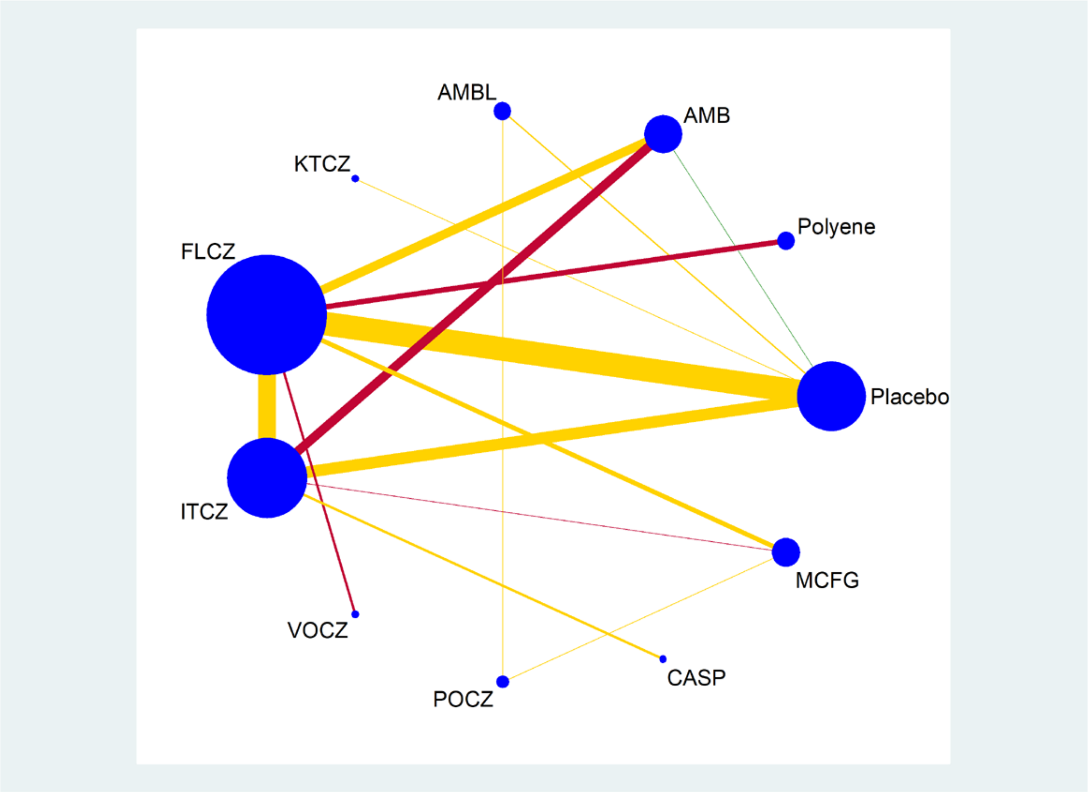

Legend: the blue nodes represent the interventions compared while the edges represent the direct comparisons available between pairs of interventions. The node sizes are weighted based on the number of patients in each arm while the edges are weighted using inverse variance. The edge colors (green or red) are weighted based on the quality of the studies included. A green edge indicates the comparison with high quality studies while a red line indicates comparison with low quality studies. AMB: conventional amphotericin B; AMBL: liposomal amphotericin B; KTCZ: ketoconazole; FLCZ: fluconazole; ITCZ: itraconazole; VOCZ: voriconazole; POCZ: posaconazole; CASP: caspofungin; MCFG: micafungin.

© 2020 Wang J et al. *JAMA Netw Open.*

Panel D.	Invasive Candidiasis

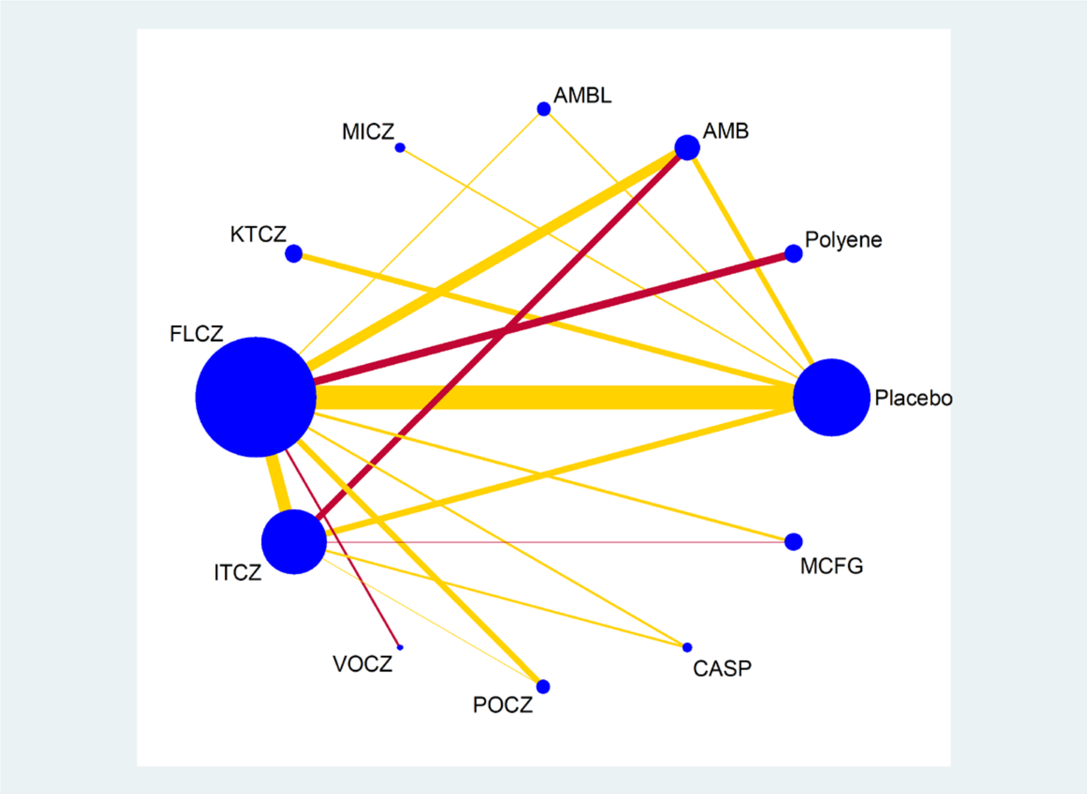

Legend: the blue nodes represent the interventions compared while the edges represent the direct comparisons available between pairs of interventions. The node sizes are weighted based on the number of patients in each arm while the edges are weighted using inverse variance. The edge colors (green or red) are weighted based on the quality of the studies included. A green edge indicates the comparison with high quality studies while a red line indicates comparison with low quality studies. AMB: conventional amphotericin B; AMBL: liposomal amphotericin B; MICZ: miconazole; KTCZ: ketoconazole; FLCZ: fluconazole; ITCZ: itraconazole; VOCZ: voriconazole; POCZ: posaconazole; CASP: caspofungin; MCFG: micafungin.

© 2020 Wang J et al. *JAMA Netw Open.*

Panel E.   Invasive Aspergillus

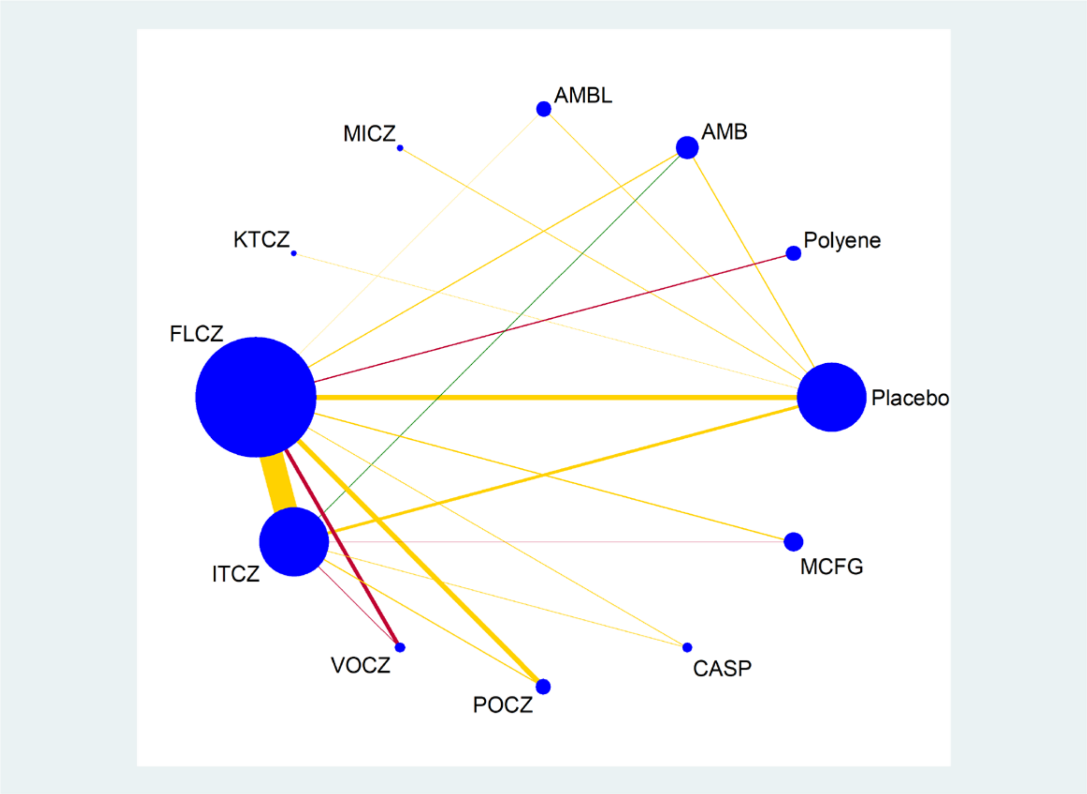

Legend: the blue nodes represent the interventions compared while the edges represent the direct comparisons available between pairs of interventions. The node sizes are weighted based on the number of patients in each arm while the edges are weighted using inverse variance. The edge colors (green or red) are weighted based on the quality of the studies included. A green edge indicates the comparison with high quality studies while a red line indicates comparison with low quality studies. AMB: conventional amphotericin B; AMBL: liposomal amphotericin B; MICZ: miconazole; KTCZ: ketoconazole; FLCZ: fluconazole; ITCZ: itraconazole; VOCZ: voriconazole; POCZ: posaconazole; CASP: caspofungin; MCFG: micafungin.

© 2020 Wang J et al. *JAMA Netw Open.*

Panel F. Mortality

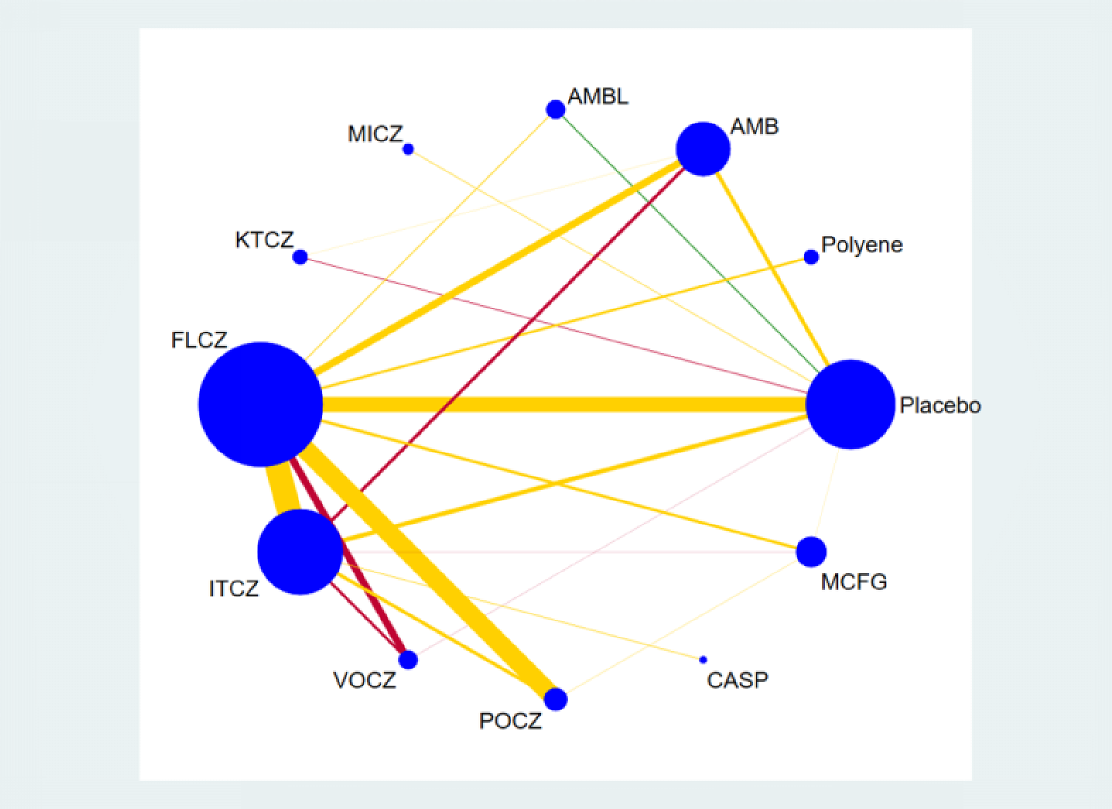

Legend: the blue nodes represent the interventions compared while the edges represent the direct comparisons available between pairs of interventions. The node sizes are weighted based on the number of patients in each arm while the edges are weighted using inverse variance. The edge colors (green or red) are weighted based on the quality of the studies included. A green edge indicates the comparison with high quality studies while a red line indicates comparison with low quality studies. AMB: conventional amphotericin B; AMBL: liposomal amphotericin B; MICZ: miconazole; KTCZ: ketoconazole; FLCZ: fluconazole; ITCZ: itraconazole; VOCZ: voriconazole; POCZ: posaconazole; CASP: caspofungin; MCFG: micafungin.

© 2020 Wang J et al. *JAMA Netw Open.*

Panel G. Fungi-related death

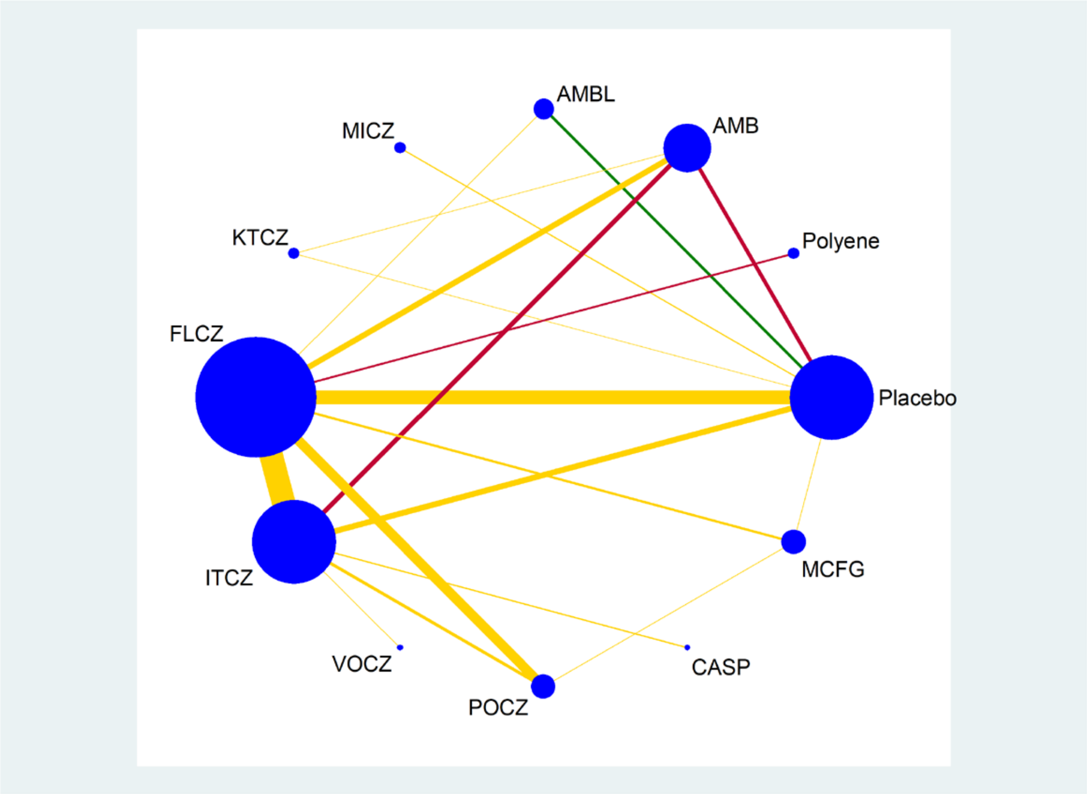

Legend: the blue nodes represent the interventions compared while the edges represent the direct comparisons available between pairs of interventions. The node sizes are weighted based on the number of patients in each arm while the edges are weighted using inverse variance. The edge colors (green or red) are weighted based on the quality of the studies included. A green edge indicates the comparison with high quality studies while a red line indicates comparison with low quality studies. AMB: conventional amphotericin B; AMBL: liposomal amphotericin B; MICZ: miconazole; KTCZ: ketoconazole; FLCZ: fluconazole; ITCZ: itraconazole; VOCZ: voriconazole; POCZ: posaconazole; CASP: caspofungin; MCFG: micafungin.

© 2020 Wang J et al. *JAMA Netw Open.*

Panel H. Withdrawal

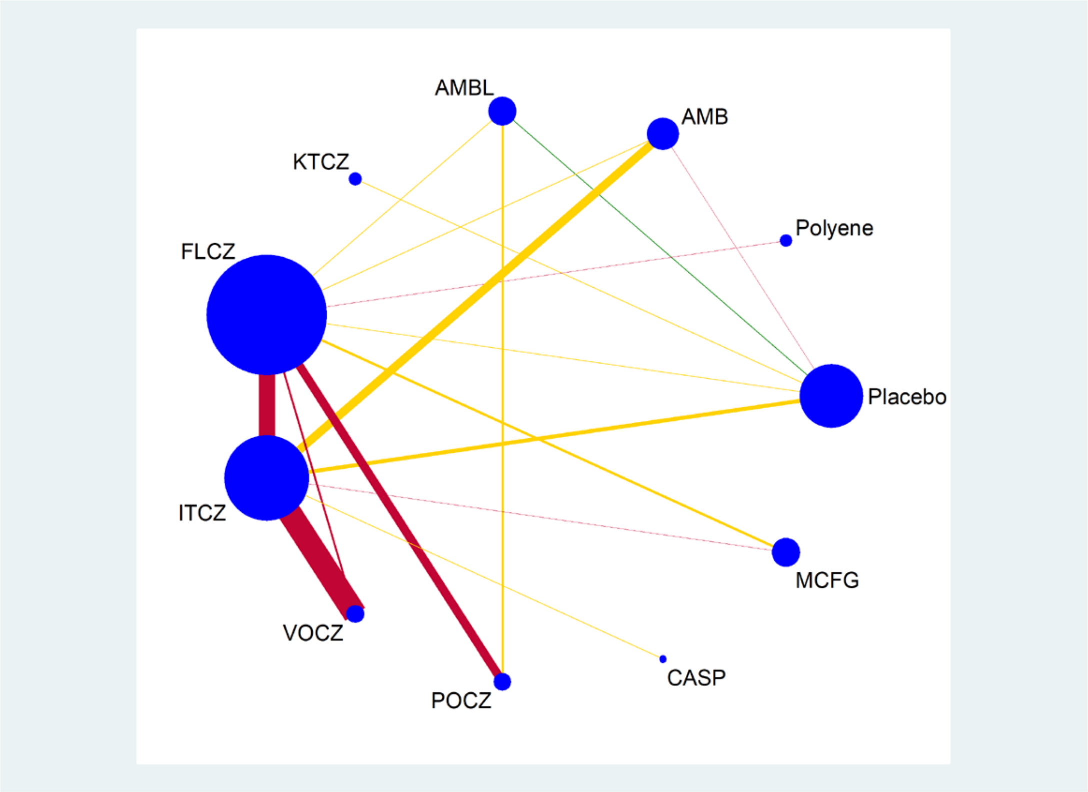

Legend: the blue nodes represent the interventions compared while the edges represent the direct comparisons available between pairs of interventions. The node sizes are weighted based on the number of patients in each arm while the edges are weighted using inverse variance. The edge colors (green or red) are weighted based on the quality of the studies included. A green edge indicates the comparison with high quality studies while a red line indicates comparison with low quality studies. AMB: conventional amphotericin B; AMBL: liposomal amphotericin B; KTCZ: ketoconazole; FLCZ: fluconazole; ITCZ: itraconazole; VOCZ: voriconazole; POCZ: posaconazole; CASP: caspofungin; MCFG: micafungin.

© 2020 Wang J et al. *JAMA Netw Open.*

eFigure 4. Forest Plots of All Outcomes

Panel A. Fungal Infections

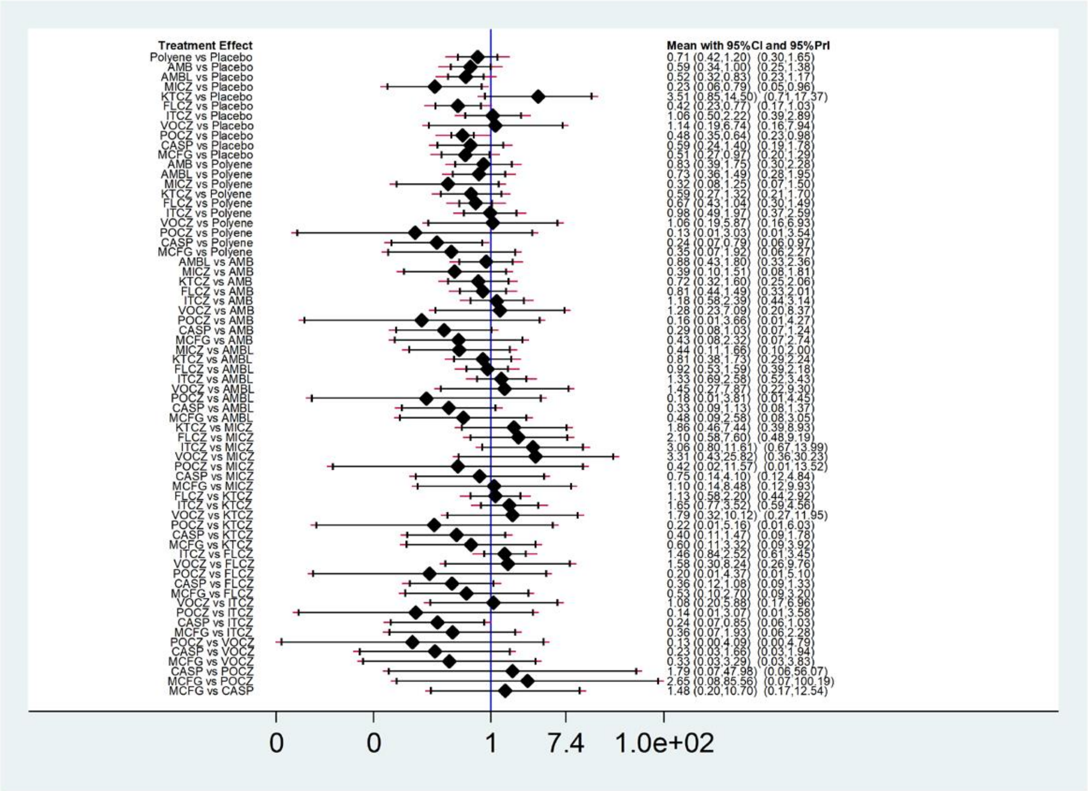

Legend: The results are reported as Relative risks (RRs) and 95% confidence intervals (CIs). The blue line (line of null effect) is equal to 1. The solid black lines represent the CIs while the diamond summarizes the RRs. For each pairwise comparison, the forest plot should be read as following: if the diamond with the entire CI did not reach the blue line of null effect, there is a significant difference. If the entire CI is on the left of the null effect, the event is significantly higher in the “intervention arm” while, when the entire CI is on the right, the event is statistically more frequent in the “reference arm”. When the entire CI crosses the line of null effect, the difference between the two procedures compared is not statistically significant. In addition, a red line reports the Predictive Interval (PrI), namely the interval within which the estimate of a future study is expected to be. AMB: conventional amphotericin B; AMBL: liposomal amphotericin B; MICZ: miconazole; KTCZ: ketoconazole; FLCZ: fluconazole; ITCZ: itraconazole; VOCZ: voriconazole; POCZ: posaconazole; CASP: caspofungin; MCFG: micafungin.

© 2020 Wang J et al. *JAMA Netw Open.*

Panel B. IFI

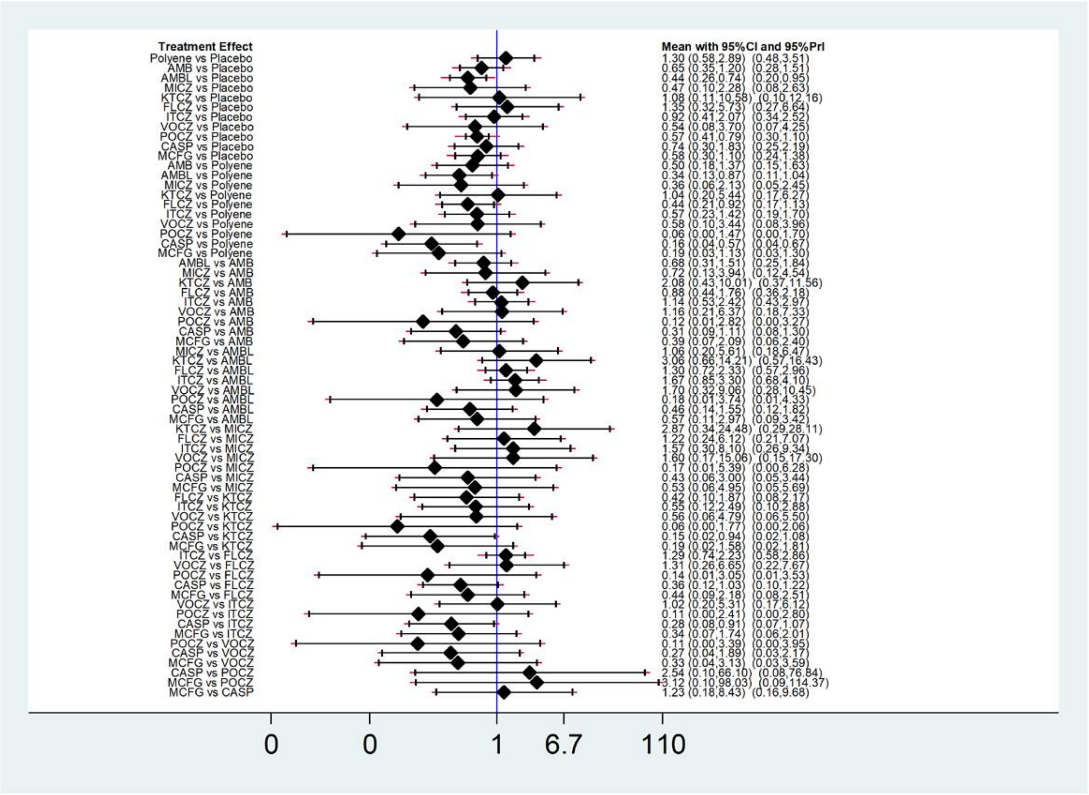

Legend: The results are reported as Relative risks (RRs) and 95% confidence intervals (CIs). The blue line (line of null effect) is equal to 1. The solid black lines represent the CIs while the diamond summarizes the RRs. For each pairwise comparison, the forest plot should be read as following: if the diamond with the entire CI did not reach the blue line of null effect, there is a significant difference. If the entire CI is on the left of the null effect, the event is significantly higher in the "intervention arm" while, when the entire CI is on the right, the event is statistically more frequent in the "reference arm". When the entire CI crosses the line of null effect, the difference between the two procedures compared is not statistically significant. In addition, a red line reports the Predictive Interval (PrI), namely the interval within which the estimate of a future study is expected to be. AMB: conventional amphotericin B; AMBL: liposomal amphotericin B; MICZ: miconazole; KTCZ: ketoconazole; FLCZ: fluconazole; ITCZ: itraconazole; VOCZ: voriconazole; POCZ: posaconazole; CASP: caspofungin; MCFG: micafungin.

Panel C. Proven IFI

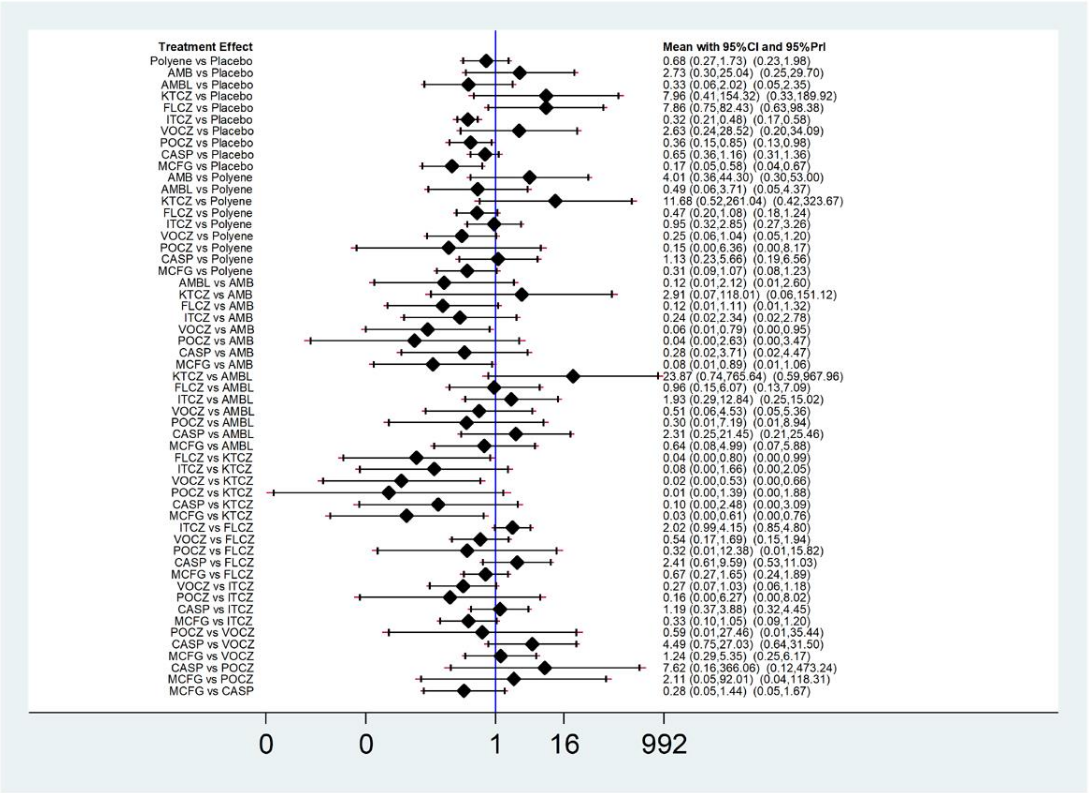

Legend: The results are reported as Relative risks (RRs) and 95% confidence intervals (CIs). The blue line (line of null effect) is equal to 1. The solid black lines represent the CIs while the diamond summarizes the RRs. For each pairwise comparison, the forest plot should be read as following: if the diamond with the entire CI did not reach the blue line of null effect, there is a significant difference. If the entire CI is on the left of the null effect, the event is significantly higher in the "intervention arm" while, when the entire CI is on the right, the event is statistically more frequent in the "reference arm". When the entire CI crosses the line of null effect, the difference between the two procedures compared is not statistically significant. In addition, a red line reports the Predictive Interval (PrI), namely the interval within which the estimate of a future study is expected to be. AMB: conventional amphotericin B; AMBL: liposomal amphotericin B; KTCZ: ketoconazole; FLCZ: fluconazole; ITCZ: itraconazole; VOCZ: voriconazole; POCZ: posaconazole; CASP: caspofungin; MCFG: micafungin.

Panel D. Invasive Candidiasis

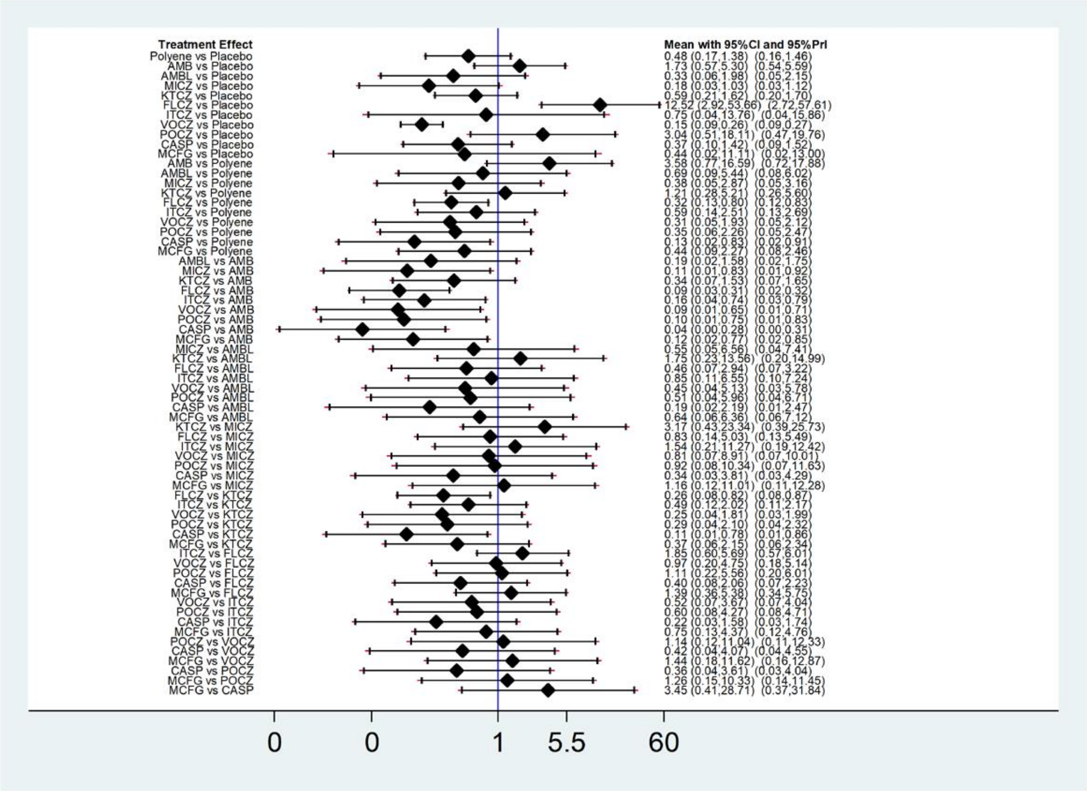

Legend: The results are reported as Relative risks (RRs) and 95% confidence intervals (CIs). The blue line (line of null effect) is equal to 1. The solid black lines represent the CIs while the diamond summarizes the RRs. For each pairwise comparison, the forest plot should be read as following: if the diamond with the entire CI did not reach the blue line of null effect, there is a significant difference. If the entire CI is on the left of the null effect, the event is significantly higher in the "intervention arm" while, when the entire CI is on the right, the event is statistically more frequent in the "reference arm". When the entire CI crosses the line of null effect, the difference between the two procedures compared is not statistically significant. In addition, a red line reports the Predictive Interval (PrI), namely the interval within which the estimate of a future study is expected to be. AMB: conventional amphotericin B; AMBL: liposomal amphotericin B; MICZ: miconazole; KTCZ: ketoconazole; FLCZ: fluconazole; ITCZ: itraconazole; VOCZ: voriconazole; POCZ: posaconazole; CASP: caspofungin; MCFG: micafungin.

© 2020 Wang J et al. *JAMA Netw Open.*

Panel E. Invasive Aspergillus

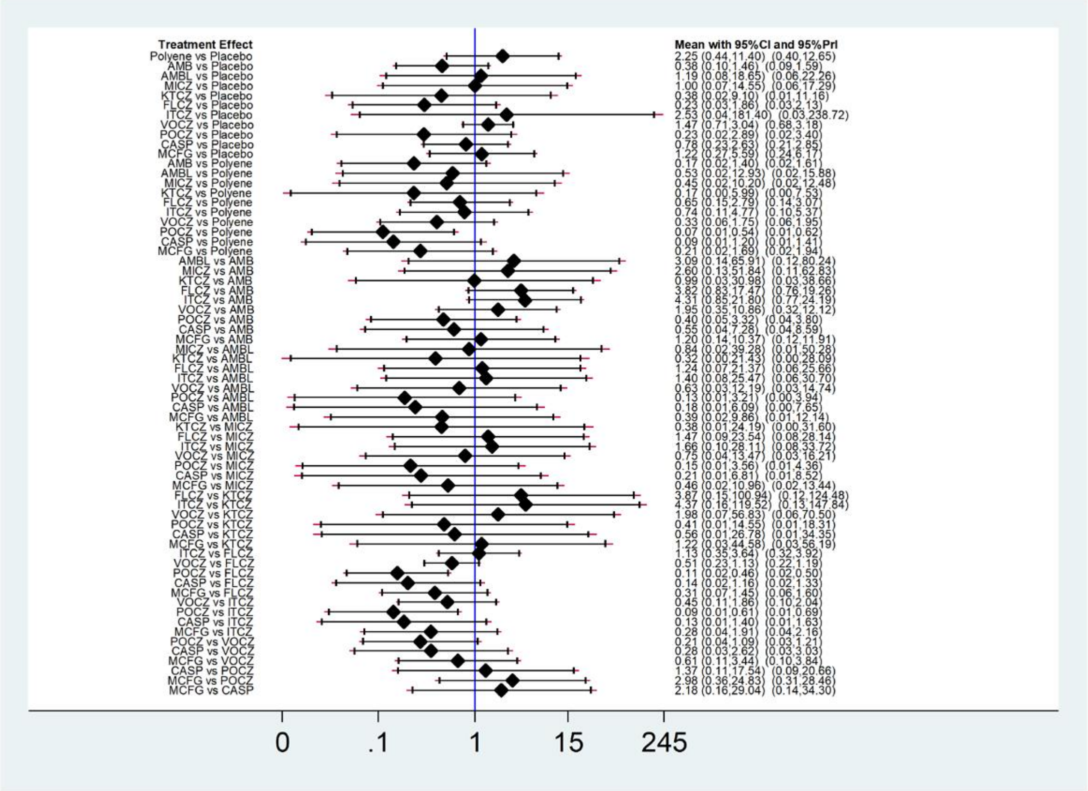

Legend: The results are reported as Relative risks (RRs) and 95% confidence intervals (CIs). The blue line (line of null effect) is equal to 1. The solid black lines represent the CIs while the diamond summarizes the RRs. For each pairwise comparison, the forest plot should be read as following: if the diamond with the entire CI did not reach the blue line of null effect, there is a significant difference. If the entire CI is on the left of the null effect, the event is significantly higher in the "intervention arm" while, when the entire CI is on the right, the event is statistically more frequent in the "reference arm". When the entire CI crosses the line of null effect, the difference between the two procedures compared is not statistically significant. In addition, a red line reports the Predictive Interval (PrI), namely the interval within which the estimate of a future study is expected to be. AMB: conventional amphotericin B; AMBL: liposomal amphotericin B; MICZ: miconazole; KTCZ: ketoconazole; FLCZ: fluconazole; ITCZ: itraconazole; VOCZ: voriconazole; POCZ: posaconazole; CASP: caspofungin; MCFG: micafungin.

© 2020 Wang J et al. *JAMA Netw Open.*

Panel F. Mortality

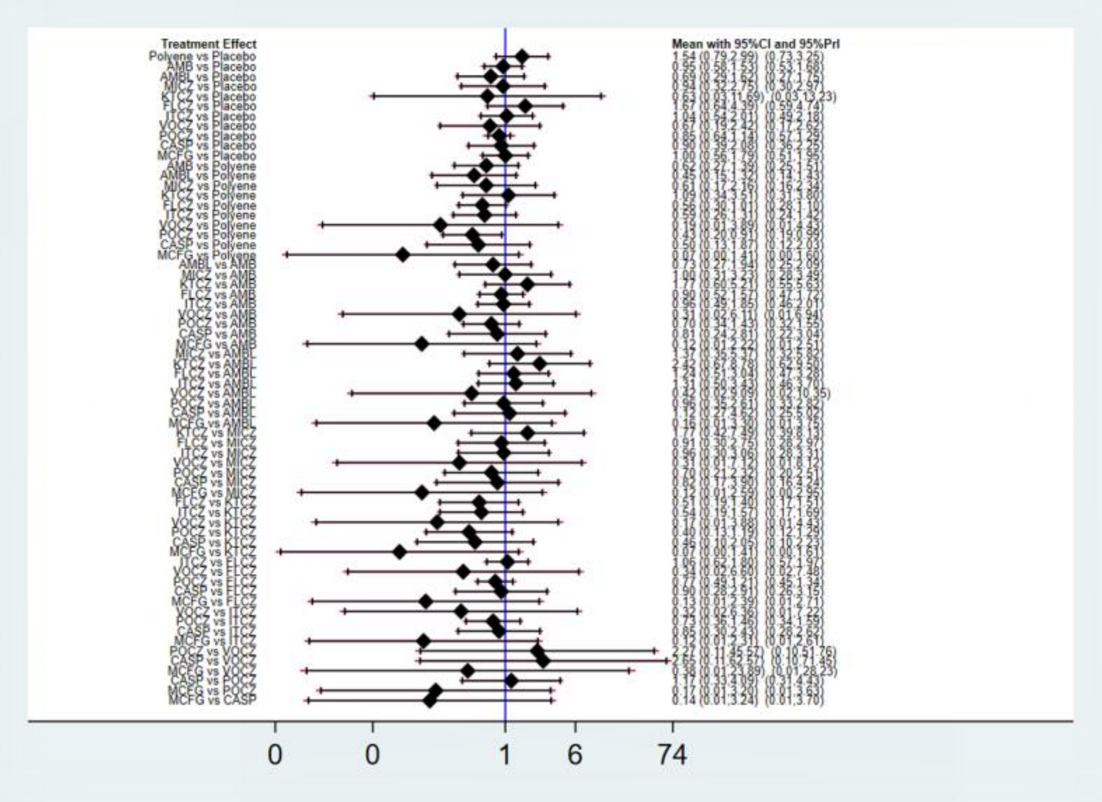

Legend: The results are reported as Relative risks (RRs) and 95% confidence intervals (CIs). The blue line (line of null effect) is equal to 1. The solid black lines represent the CIs while the diamond summarizes the RRs. For each pairwise comparison, the forest plot should be read as following: if the diamond with the entire CI did not reach the blue line of null effect, there is a significant difference. If the entire CI is on the left of the null effect, the event is significantly higher in the “intervention arm” while, when the entire CI is on the right, the event is statistically more frequent in the “reference arm”. When the entire CI crosses the line of null effect, the difference between the two procedures compared is not statistically significant. In addition, a red line reports the Predictive Interval (PrI), namely the interval within which the estimate of a future study is expected to be. AMB: conventional amphotericin B; AMBL: liposomal amphotericin B; MICZ: miconazole; KTCZ: ketoconazole; FLCZ: fluconazole; ITCZ: itraconazole; VOCZ: voriconazole; POCZ: posaconazole; CASP: caspofungin; MCFG: micafungin.

Panel G. Fungi-related death

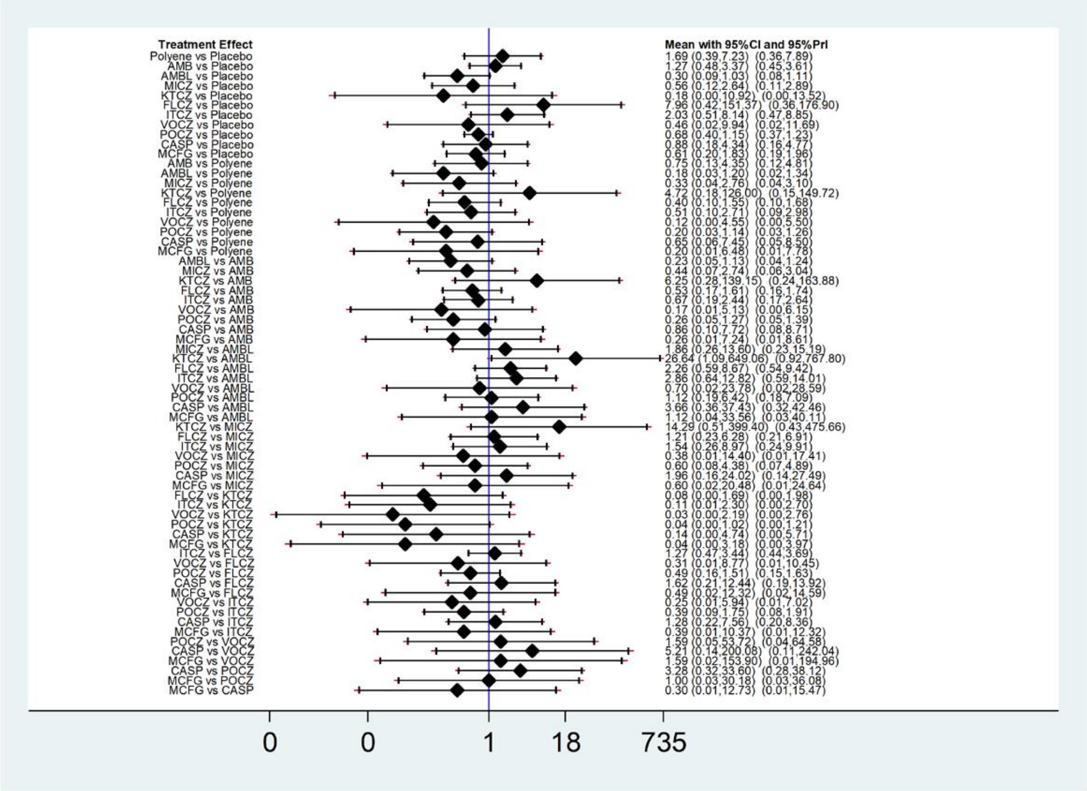

Legend: The results are reported as Relative risks (RRs) and 95% confidence intervals (CIs). The blue line (line of null effect) is equal to 1. The solid black lines represent the CIs while the diamond summarizes the RRs. For each pairwise comparison, the forest plot should be read as following: if the diamond with the entire CI did not reach the blue line of null effect, there is a significant difference. If the entire CI is on the left of the null effect, the event is significantly higher in the "intervention arm" while, when the entire CI is on the right, the event is statistically more frequent in the "reference arm". When the entire CI crosses the line of null effect, the difference between the two procedures compared is not statistically significant. In addition, a red line reports the Predictive Interval (PrI), namely the interval within which the estimate of a future study is expected to be. AMB: conventional amphotericin B; AMBL: liposomal amphotericin B; MICZ: miconazole; KTCZ: ketoconazole; FLCZ: fluconazole; ITCZ: itraconazole; VOCZ: voriconazole; POCZ: posaconazole; CASP: caspofungin; MCFG: micafungin.

© 2020 Wang J et al. *JAMA Netw Open.*

Panel H. Withdrawal

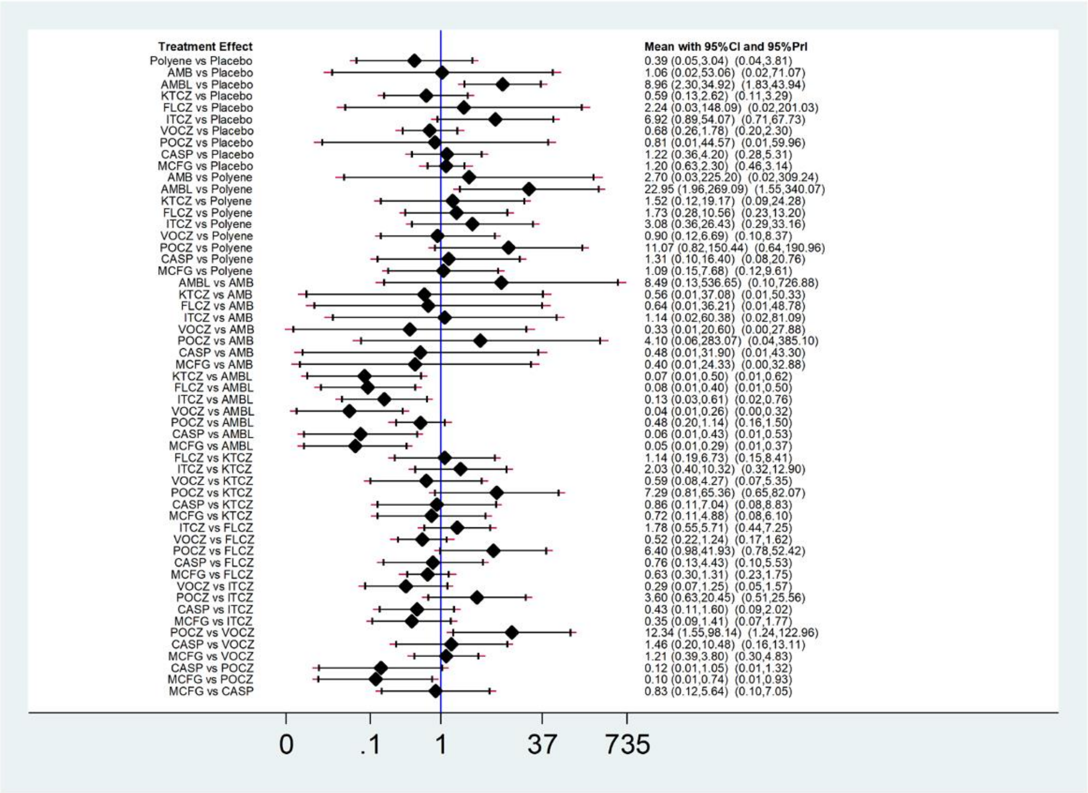

Legend: The results are reported as Relative risks (RRs) and 95% confidence intervals (CIs). The blue line (line of null effect) is equal to 1. The solid black lines represent the CIs while the diamond summarizes the RRs. For each pairwise comparison, the forest plot should be read as following: if the diamond with the entire CI did not reach the blue line of null effect, there is a significant difference. If the entire CI is on the left of the null effect, the event is significantly higher in the "intervention arm" while, when the entire CI is on the right, the event is statistically more frequent in the "reference arm". When the entire CI crosses the line of null effect, the difference between the two procedures compared is not statistically significant. In addition, a red line reports the Predictive Interval (PrI), namely the interval within which the estimate of a future study is expected to be. AMB: conventional amphotericin B; AMBL: liposomal amphotericin B; KTCZ: ketoconazole; FLCZ: fluconazole; ITCZ: itraconazole; VOCZ: voriconazole; POCZ: posaconazole; CASP: caspofungin; MCFG: micafungin.

© 2020 Wang J et al. *JAMA Netw Open.*

eFigure 5. League Tables of All Outcomes

Panel A. Fungal Infections

**Placebo**												
**0.51**<br>**(0.27,0.97)**	**MCFG**											
0.59<br>(0.24,1.40)	0.68<br>(0.09,4.89)	**CASP**										
**0.48**<br>**(0.35,0.64)**	0.38<br>(0.01,12.17)	0.56<br>(0.02,14.92)	**POCZ**									
1.14<br>(0.19,6.74)	3.00<br>(0.30,29.57)	4.44<br>(0.60,32.58)	7.95<br>(0.24,258.87)	**VOCZ**								
1.06<br>(0.50,2.22)	2.77<br>(0.52,14.76)	**4.09**<br>**(1.18,14.17)**	7.34<br>(0.33,165.09)	0.92<br>(0.17,5.01)	**ITCZ**							
0.42<br>(0.23,0.77)	1.90<br>(0.37,9.75)	2.81<br>(0.92,8.57)	5.04<br>(0.23,111.00)	0.63<br>(0.12,3.31)	0.69<br>(0.40,1.19)	**FLCZ**						
3.51<br>(0.85,14.50)	1.68<br>(0.30,9.36)	2.48<br>(0.68,9.10)	4.45<br>(0.19,102.44)	0.56<br>(0.10,3.17)	0.61<br>(0.28,1.30)	0.88<br>(0.45,1.72)	**KTCZ**					
**0.23**<br>**(0.06,0.79)**	0.91<br>(0.12,6.95)	1.34<br>(0.24,7.34)	2.40<br>(0.09,66.64)	0.30<br>(0.04,2.35)	0.33<br>(0.09,1.24)	0.48<br>(0.13,1.72)	0.54<br>(0.13,2.16)	**MICZ**				
**0.52**<br>**(0.32,0.83)**	2.07<br>(0.39,11.09)	3.07<br>(0.89,10.63)	5.50<br>(0.26,115.32)	0.69<br>(0.13,3.76)	0.75<br>(0.39,1.45)	1.09<br>(0.63,1.89)	1.23<br>(0.58,2.63)	2.29<br>(0.60,8.73))	**AMBL**			
**0.59**<br>**(0.34,1.00)**	2.35<br>(0.43,12.77)	3.47<br>(0.97,12.35)	6.22<br>(0.27,141.48)	0.78<br>(0.14,4.33)	0.85<br>(0.42,1.72)	1.23<br>(0.67,2.27)	1.40<br>(0.63,3.12)	2.59<br>(0.66,10.10)	1.13<br>(0.56,2.30)	**AMB**		
0.71<br>(0.42,1.20)	2.83<br>(0.52,15.36)	**4.18**<br>**(1.26,13.85)**	7.49<br>(0.33,170.31)	0.94<br>(0.17,5.21)	1.02<br>(0.51,2.06)	1.49<br>(0.96,2.31)	1.68<br>(0.76,3.74)	3.12<br>(0.80,12.14)	1.36<br>(0.67,2.76)	1.21<br>(0.57,2.55)	**Polyene**	

Legend: RR >1 means the treatment in top left is better. AMB: conventional amphotericin B; AMBL: liposomal amphotericin B; MICZ: miconazole; KTCZ: ketoconazole; FLCZ: fluconazole; ITCZ: itraconazole; VOCZ: voriconazole; POCZ: posaconazole; CASP: caspofungin; MCFG: micafungin.

© 2020 Wang J et al. *JAMA Netw Open.*

Panel B. IFI

**Placebo**												
0.58 (0.30,1.10)	**MCFG**											
0.74 (0.30,1.83)	0.81 (0.12,5.59)	**CASP**										
**0.57 (0.42,0.79)**	0.32 (0.01,10.07)	0.39 (0.02,10.24)	**POCZ**									
0.54 (0.08,3.70)	3.00 (0.32,28.14)	3.68 (0.53,25.67)	9.36 (0.30,296.64)	**VOCZ**								
0.92 (0.41,2.07)	2.95 (0.58,15.10)	**3.62 (1.09,11.98)**	9.19 (0.41,204.06)	0.98 (0.19,5.13)	**ITCZ**							
1.35 (0.32,5.73)	2.29 (0.46,11.41)	2.81 (0.97,8.14)	7.14 (0.33,155.48)	0.76 (0.15,3.87)	0.78 (0.45,1.35)	**FLCZ**						
1.08 (0.11,10.58)	5.39 (0.63,45.72)	**6.62 (1.07,41.06)**	16.81 (0.57,499.89)	1.80 (0.21,15.45)	1.83 (0.40,8.30)	2.35 (0.53,10.38)	**KTCZ**					
0.47 (0.10,2.28)	1.88 (0.20,17.45)	2.30 (0.33,15.91)	5.85 (0.19,184.60)	0.63 (0.07,5.89)	0.64 (0.12,3.28)	0.82 (0.16,4.11)	0.35 (0.04,2.97)	**MICZ**				
**0.44 (0.26,0.74)**	1.76 (0.34,9.22)	2.16 (0.64,7.28)	5.50 (0.27,113.20)	0.59 (0.11,3.13)	0.60 (0.30,1.18)	0.77 (0.43,1.38)	0.33 (0.07,1.52)	0.94 (0.18,4.95)	**AMBL**			
0.65 (0.35,1.20)	2.60 (0.48,14.05)	3.19 (0.90,11.32)	8.10 (0.35,184.78)	0.87 (0.16,4.77)	0.88 (0.41,1.88)	1.13 (0.57,2.26)	0.48 (0.10,2.32)	1.38 (0.25,7.54)	1.47 (0.66,3.26)	**AMB**		
1.30 (0.58,2.89)	5.18 (0.89,30.29)	**6.37 (1.75,23.18)**	16.17 (0.68,383.87)	1.73 (0.29,10.27)	1.76 (0.70,4.39)	**2.26 (1.09,4.72)**	0.96 (0.18,5.04)	2.76 (0.47,16.25)	**2.94 (1.15,7.52)**	2.00 (0.73,5.46)	**Polyene**	

Legend: RR >1 means the treatment in top left is better. AMB: conventional amphotericin B; AMBL: liposomal amphotericin B; MICZ: miconazole; KTCZ: ketoconazole; FLCZ: fluconazole; ITCZ: itraconazole; VOCZ: voriconazole; POCZ: posaconazole; CASP: caspofungin; MCFG: micafungin.

© 2020 Wang J et al. *JAMA Netw Open.*

Panel C. Proven IFI

**Placebo**											
**0.17 (0.05,0.58)**	**MCFG**										
0.65 (0.36,1.16)	3.61 (0.69,18.75)	**CASP**									
**0.36 (0.15,0.85)**	0.47 (0.01,20.66)	0.13 (0.00,6.31)	**POCZ**								
2.63 (0.24,28.52)	0.80 (0.19,3.46)	0.22 (0.04,1.34)	1.70 (0.04,79.09)	**VOCZ**							
**0.32 (0.21,0.48)**	3.03 (0.96,9.57)	0.84 (0.26,2.72)	6.39 (0.16,255.44)	3.76 (0.97,14.58)	**ITCZ**						
7.86 (0.75,82.43)	1.50 (0.61,3.69)	0.41 (0.10,1.65)	3.16 (0.08,123.47)	1.86 (0.59,5.87)	0.49 (0.24,1.01)	**FLCZ**					
7.96 (0.41,154.32)	**37.33 (1.64,850.80**	10.34 (0.40,265.04)	78.77 (0.72,8627.55)	**46.42 (1.88,1145.92)**	12.34 (0.60,253.24)	**24.94 (1.25,497.77)**	**KTCZ**				
NA	NA	NA	NA	NA	NA	NA	NA	**MICZ**			
0.33 (0.06,2.02)	1.56 (0.20,12.22)	0.43 (0.05,4.03)	3.30 (0.14,78.29)	1.94 (0.22,17.12)	0.52 (0.08,3.43)	1.04 (0.16,6.63)	0.04 (0.00,1.34)	NA	**AMBL**		
2.73 (0.30,25.04)	**12.81 (1.13,145.20)**	3.55 (0.27,46.70)	27.02 (0.38,1919.88)	**15.92 (1.27,199.94)**	4.23 (0.43,41.87)	8.56 (0.90,81.56)	0.34 (0.01,13.89)	NA	8.19 (0.47,142.23)	**AMB**	
0.68 (0.27,1.73)	3.20 (0.94,10.91)	0.89 (0.18,4.44)	6.74 (0.16,289.39)	3.97 (0.96,16.40)	1.06 (0.35,3.17)	2.14 (0.93,4.90)	0.09 (0.00,1.91)	NA	2.04 (0.27,15.50)	0.25 (0.02,2.76)	**Polyene**

Legend: RR >1 means the treatment in top left is better. AMB: conventional amphotericin B; AMBL: liposomal amphotericin B; MICZ: miconazole; KTCZ: ketoconazole; FLCZ: fluconazole; ITCZ: itraconazole; VOCZ: voriconazole; POCZ: posaconazole; CASP: caspofungin; MCFG: micafungin.

© 2020 Wang J et al. *JAMA Netw Open.*

Panel D. Invasive Candidiasis

**Placebo**											
0.44 (0.02,11.11)	**MCFG**										
0.37 (0.10,1.42)	0.29 (0.03,2.41)	**CASP**									
3.04 (0.51,18.11)	0.80 (0.10,6.53)	2.74 (0.28,27.22)	**POCZ**								
**0.15 (0.09,0.26)**	0.70 (0.09,5.62)	2.40 (0.25,23.43)	0.87 (0.09,8.44)	**VOCZ**							
0.75 (0.04,13.76)	1.33 (0.23,7.71)	4.59 (0.63,33.22)	1.67 (0.23,11.94)	1.91 (0.27,13.42)	**ITCZ**						
**12.52 (2.92,53.66)**	0.72 (0.19,2.78)	2.48 (0.49,12.67)	0.90 (0.18,4.54)	1.03 (0.21,5.08)	0.54 (0.18,1.66)	**FLCZ**					
0.59 (0.21,1.62)	2.73 (0.47,15.99)	**9.41 (1.29,68.78)**	3.43 (0.48,24.73)	3.92 (0.55,27.79)	2.05 (0.49,8.51)	**3.79 (1.21,11.85)**	**KTCZ**				
0.18 (0.03,1.03)	0.86 (0.09,8.16)	2.97 (0.26,33.62)	1.08 (0.10,12.12)	1.24 (0.11,13.66)	0.65 (0.09,4.73)	0.65 (0.09,4.73)	0.32 (0.04,2.33)	**MICZ**			
0.33 (0.06,1.98)	1.56 (0.16,15.41)	5.37 (0.46,63.28)	1.96 (0.17,22.81)	2.24 (0.19,25.72)	1.17 (0.15,8.98)	2.16 (0.34,13.78)	0.57 (0.07,4.42)	1.81 (0.15,21.44)	**AMBL**		
1.73 (0.57,5.30)	**8.06 (1.29,50.27)**	**27.82 (3.60,214.81)**	**10.14 (1.33,77.27)**	**11.60 (1.55,86.85)**	**6.07 (1.36,27.14)**	**11.21 (3.27,38.47)**	2.96 (0.65,13.39)	**9.36 (1.20,72.88)**	5.18 (0.63,42.30)	**AMB**	
0.48 (0.17,1.38)	2.25 (0.44,11.53)	**7.78 (1.20,50.40)**	2.83 (0.44,18.11)	3.24 (0.52,20.32)	1.70 (0.40,7.21)	**3.13 (1.26,7.82)**	0.83 (0.19,3.56)	2.62 (0.35,19.64)	1.45 (0.18,11.41)	0.28 (0.06,1.30)	**Polyene**

Legend: RR >1 means the treatment in top left is better. AMB: conventional amphotericin B; AMBL: liposomal amphotericin B; MICZ: miconazole; KTCZ: ketoconazole; FLCZ: fluconazole; ITCZ: itraconazole; VOCZ: voriconazole; POCZ: posaconazole; CASP: caspofungin; MCFG: micafungin.

© 2020 Wang J et al. *JAMA Netw Open.*

Panel E. Invasive Aspergillus

**Placebo**											
1.22 (0.27,5.59)	**MCFG**										
0.78 (0.23,2.63)	0.46 (0.03,6.12)	**CASP**									
0.23 (0.02,2.89)	0.34 (0.04,2.80)	0.73 (0.06,9.37)	**POCZ**								
1.47 (0.71,3.04)	1.63 (0.29,9.16)	3.56 (0.38,33.18)	4.86 (0.92,25.74)	**VOCZ**							
2.53 (0.04,181.40)	3.60 (0.52,24.74)	7.84 (0.72,85.87)	**10.72 (1.64,69.97)**	2.20 (0.54,9.06)	**ITCZ**						
0.23 (0.03,1.86)	3.19 (0.69,14.75)	6.95 (0.86,56.04)	**9.50 (2.19,41.14)**	1.95 (0.88,4.31)	0.89 (0.27,2.86)	**FLCZ**					
0.38 (0.02,9.10)	0.82 (0.02,30.19)	1.79 (0.04,86.11)	2.45 (0.07,87.51)	0.50 (0.02,14.44)	0.23 (0.01,6.25)	0.26 (0.01,6.73)	**KTCZ**				
1.00 (0.07,14.55)	2.17 (0.09,51.66)	4.73 (0.15,152.38)	6.47 (0.28,149.16)	1.33 (0.07,23.82)	0.60 (0.04,10.24)	0.68 (0.04,10.92)	2.64 (0.04,168.36)	**MICZ**			
1.19 (0.08,18.65)	2.58 (0.10,65.43)	5.61 (0.16,191.94)	7.68 (0.31,189.06)	1.58 (0.08,30.36)	0.72 (0.04,13.06)	0.81 (0.05,13.95)	3.13 (0.05,210.02)	1.19 (0.03,55.31)	**AMBL**		
0.38 (0.10,1.46)	0.83 (0.10,7.23)	1.82 (0.14,24.08)	2.49 (0.30,20.57)	0.51 (0.09,2.84)	0.23 (0.05,1.17)	0.26 (0.06,1.20)	1.01 (0.03,31.89)	**0.38 (0.02,7.67)**	0.32 (0.02,6.92)	**AMB**	
2.25 (0.44,11.40)	4.88 (0.59,40.27)	10.62 (0.83,135.19)	**14.53 (1.85,114.41)**	2.99 (0.57,15.62)	1.36 (0.21,8.76)	1.53 (0.36,6.54)	5.92 (0.17,210.28)	2.25 (0.10,51.47)	1.89 (0.08,46.33)	5.84 (0.71,47.83)	**Polyene**

Legend: RR >1 means the treatment in top left is better. AMB: conventional amphotericin B; AMBL: liposomal amphotericin B; MICZ: miconazole; KTCZ: ketoconazole; FLCZ: fluconazole; ITCZ: itraconazole; VOCZ: voriconazole; POCZ: posaconazole; CASP: caspofungin; MCFG: micafungin.

© 2020 Wang J et al. *JAMA Netw Open.*

Panel F. Mortality

**Placebo**												
1.00 (0.56,1.78)	**MCFG**											
1.11 (0.48,2.56)	0.14 (0.01,3.24)	**CASP**										
1.17 (0.88,1.56)	0.17 (0.01,3.20)	1.17 (0.33,4.09)	**POCZ**									
1.48 (0.41,5.32)	0.38 (0.01,23.89)	2.65 (0.11,62.57)	2.27 (0.11,45.57)	**VOCZ**								
0.96 (0.50,1.87)	0.12 (0.01,2.31)	0.85 (0.30,2.43)	0.73 (0.36,1.46)	0.32 (0.02,6.36)	**ITCZ**							
0.60 (0.23,1.57)	0.13 (0.01,2.39)	0.90 (0.28,2.91)	0.77 (0.49,1.21)	0.34 (0.02,6.60)	1.06 (0.62,1.80)	**FLCZ**						
1.58 (0.09,29.16)	0.07 (0.00,1.41)	0.46 (0.10,2.05)	0.40 (0.13,1.19)	0.17 (0.01,3.88)	0.54 (0.19,1.57)	0.51 (0.19,1.40)	**KTCZ**					
1.06 (0.36,3.09)	0.12 (0.01,2.59)	0.82 (0.17,3.90)	0.70 (0.21,2.32)	0.31 (0.01,7.12)	0.96 (0.30,3.06)	0.91 (0.30,2.75)	1.77 (0.42,7.49)	**MICZ**				
1.45 (0.62,3.39)	0.16 (0.01,3.30)	1.12 (0.27,4.62)	0.96 (0.35,2.61)	0.42 (0.02,9.09)	1.31 (0.50,3.43)	1.24 (0.51,3.04)	2.42 (0.67,8.78)	1.37 (0.35,5.37)	**AMBL**			
1.06 (0.65,1.71)	0.12 (0.01,2.22)	0.81 (0.24,2.81)	0.70 (0.34,1.43)	0.31 (0.02,6.11)	0.96 (0.49,1.85)	0.90 (0.52,1.57)	1.77 (0.60,5.21)	1.00 (0.31,3.23)	0.73 (0.27,1.94)	**AMB**		
0.65 (0.33,1.27)	0.07 (0.00,1.41)	0.50 (0.13,1.87)	0.43 (0.20,0.91)	0.19 (0.01,3.89)	0.59 (0.26,1.31)	0.56 (0.30,1.01)	1.09 (0.34,3.51)	0.61 (0.17,2.16)	0.45 (0.15,1.32)	0.62 (0.27,1.39)	**Polyene**	

Legend: RR >1 means the treatment in top left is better. AMB: conventional amphotericin B; AMBL: liposomal amphotericin B; MICZ: miconazole; KTCZ: ketoconazole; FLCZ: fluconazole; ITCZ: itraconazole; VOCZ: voriconazole; POCZ: posaconazole; CASP: caspofungin; MCFG: micafungin.

© 2020 Wang J et al. *JAMA Netw Open.*

Panel G. Fungi-related death

**Placebo**												
0.61 (0.20,1.83)	**MCFG**											
0.88 (0.18,4.34)	3.28 (0.08,137.15)	**CASP**										
0.68 (0.40,1.15)	1.00 (0.03,30.18)	0.30 (0.03,3.12)	**POCZ**									
0.46 (0.02,9.94)	0.63 (0.01,61.12)	0.19 (0.00,7.38)	0.63 (0.02,21.34)	**VOCZ**								
2.03 (0.51,8.14)	2.57 (0.10,68.42)	0.78 (0.13,4.63)	2.57 (0.57,11.52)	4.08 (0.17,98.63)	**ITCZ**							
7.96 (0.42,151.37)	2.03 (0.08,50.60)	0.62 (0.08,4.74)	2.03 (0.66,6.21)	3.22 (0.11,90.67)	0.79 (0.29,2.14)	**FLCZ**						
0.18 (0.00,10.92)	23.88 (0.31,1811.79)	7.28 (0.21,251.17)	23.88 (0.98,583.79)	37.90 (0.46,3148.74)	9.30 (0.43,198.93)	11.78 (0.59,235.20)	**KTCZ**					
0.56 (0.12,2.64)	1.67 (0.05,57.18)	0.51 (0.04,6.23)	1.67 (0.23,12.22)	2.65 (0.07,101.19)	0.65 (0.11,3.80)	0.82 (0.16,4.27)	0.07 (0.00,1.95)	**MICZ**				
0.30 (0.09,1.03)	0.90 (0.03,26.97)	0.27 (0.03,2.79)	0.90 (0.16,5.16)	1.42 (0.04,48.12)	0.35 (0.08,1.56)	0.44 (0.12,1.70)	**0.04 (0.00,0.91)**	0.54 (0.07,3.92)	**AMBL**			
1.27 (0.48,3.37)	3.82 (0.14,105.52)	1.16 (0.13,10.46)	3.82 (0.79,18.52)	6.06 (0.19,188.47)	1.49 (0.41,5.40)	1.88 (0.62,5.73)	0.16 (0.01,3.56)	2.29 (0.36,14.33)	4.26 (0.89,20.50)	**AMB**		
1.69 (0.39,7.23)	5.06 (0.15,166.06)	1.54 (0.13,17.73)	5.06 (0.87,29.34)	8.03 (0.22,293.90)	1.97 (0.37,10.52)	2.50 (0.65,9.67)	2.50 (0.65,9.67)	3.03 (0.36,25.36).76)	5.65 (0.83,38.28)	1.33 (0.23,7.65)	**Polyene**	

Legend: RR >1 means the treatment in top left is better. AMB: conventional amphotericin B; AMBL: liposomal amphotericin B; MICZ: miconazole; KTCZ: ketoconazole; FLCZ: fluconazole; ITCZ: itraconazole; VOCZ: voriconazole; POCZ: posaconazole; CASP: caspofungin; MCFG: micafungin.

© 2020 Wang J et al. *JAMA Netw Open.*

Panel H. Withdrawal

**Placebo**												
1.20 (0.63,2.30)	**MCFG**											
1.22 (0.36,4.20)	1.20 (0.18,8.17)	**CASP**										
0.81 (0.01,44.57)	**10.17 (1.35,76.52)**	8.45 (0.95,75.15)	**POCZ**									
0.68 (0.26,1.78)	0.82 (0.26,2.58)	0.68 (0.10,4.91)	0.08 (0.01,0.64)	**VOCZ**								
6.92 (0.89,54.07)	2.83 (0.71,11.25)	2.35 (0.62,8.84)	0.28 (0.05,1.58)	3.43 (0.80,14.73)	**ITCZ**							
2.24 (0.03,148.09)	1.59 (0.76,3.31)	1.32 (0.23,7.72)	0.16 (0.02,1.02)	1.93 (0.81,4.62)	0.56 (0.18,1.81)	**FLCZ**						
0.59 (0.13,2.62)	1.39 (0.20,9.49)	1.16 (0.14,9.45)	0.14 (0.02,1.23)	1.69 (0.23,12.24)	0.49 (0.10,2.51)	0.88 (0.15,5.18)	**KTCZ**					
NA	NA	NA	NA	NA	NA	NA	NA	**MICZ**				
**8.96 (2.30,34.92)**	**21.08 (3.40,130.57)**	**17.50 (2.35,130.35)**	2.07 (0.87,4.92)	**25.58 (3.89,168.42)**	**7.46 (1.65,33.67)**	**13.26 (2.50,70.41)**	**15.11 (2.01,113.45)**	NA	**AMBL**			
1.06 (0.02,53.06)	2.48 (0.04,150.01)	2.06 (0.03,135.71)	0.24 (0.00,16.88)	3.01 (0.05,187.18)	0.88 (0.02,46.59)	1.56 (0.03,88.33)	1.78 (0.03,117.57)	NA	0.12 (0.00,7.45))	**AMB**		
0.39 (0.05,3.04)	0.92 (0.13,6.48)	0.76 (0.06,9.54)	0.09 (0.01,1.23)	1.11 (0.15,8.31)	0.32 (0.04,2.79)	0.58 (0.09,3.52)	0.66 (0.05,8.31)	NA	**0.04 (0.00,0.51)**	0.37 (0.00,30.79)	**Polyene**	

Legend: RR >1 means the treatment in top left is better. AMB: conventional amphotericin B; AMBL: liposomal amphotericin B; MICZ: miconazole; KTCZ: ketoconazole; FLCZ: fluconazole; ITCZ: itraconazole; VOCZ: voriconazole; POCZ: posaconazole; CASP: caspofungin; MCFG: micafungin.

© 2020 Wang J et al. *JAMA Netw Open.*

eFigure 6. Cluster Rank Combined the SUCRA

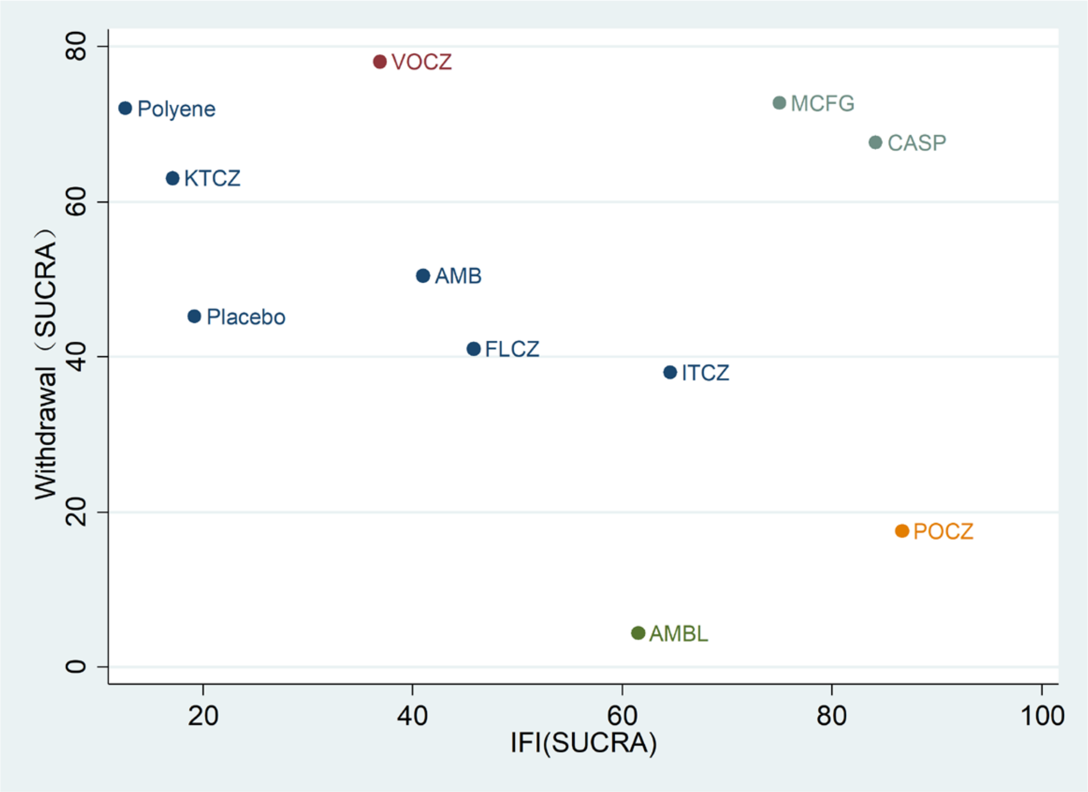

Legend: The y axis reports the SUCRA values as a percentage of "Withdrawal" . The x axis reports the IFI. Different colors identify the different clusters. AMB: conventional amphotericin B; AMBL: liposomal amphotericin B; KTCZ: ketoconazole; FLCZ: fluconazole; ITCZ: itraconazole; VOCZ: voriconazole; POCZ: posaconazole; CASP: caspofungin; MCFG: micafungin.

© 2020 Wang J et al. *JAMA Netw Open.*

eFigure 7. Inconsistency Plot of All Outcomes

Panel A. Fungal Infections

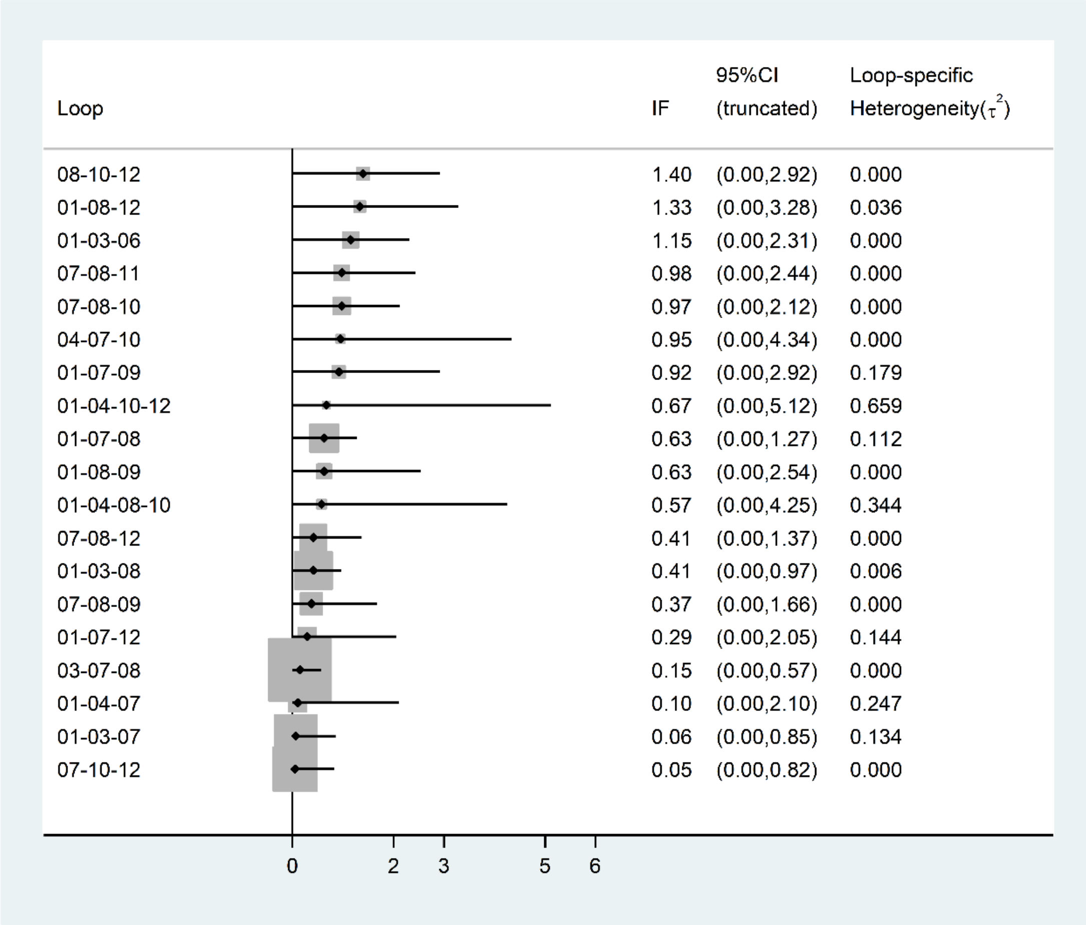

Legend: Inconsistency plot assuming loop-specific heterogeneity estimates using the method of moments estimator. arm 01= placebo; arm 02= oral polyene; arm 03= conventional amphotericin B (AMB); arm 04= liposomal amphotericin B (AMBL); arm 05= miconazole (MICZ); arm 06= ketoconazole (KTCZ); arm 07= fluconazole (FLCZ); arm 08= itraconazole (ITCZ); arm 09= voriconazole (VOCZ); arm 10= posaconazole (POCZ); arm 11= caspofungin (CASP); arm 12= micafungin (MCFG).

© 2020 Wang J et al. *JAMA Netw Open.*

Panel B. IFI

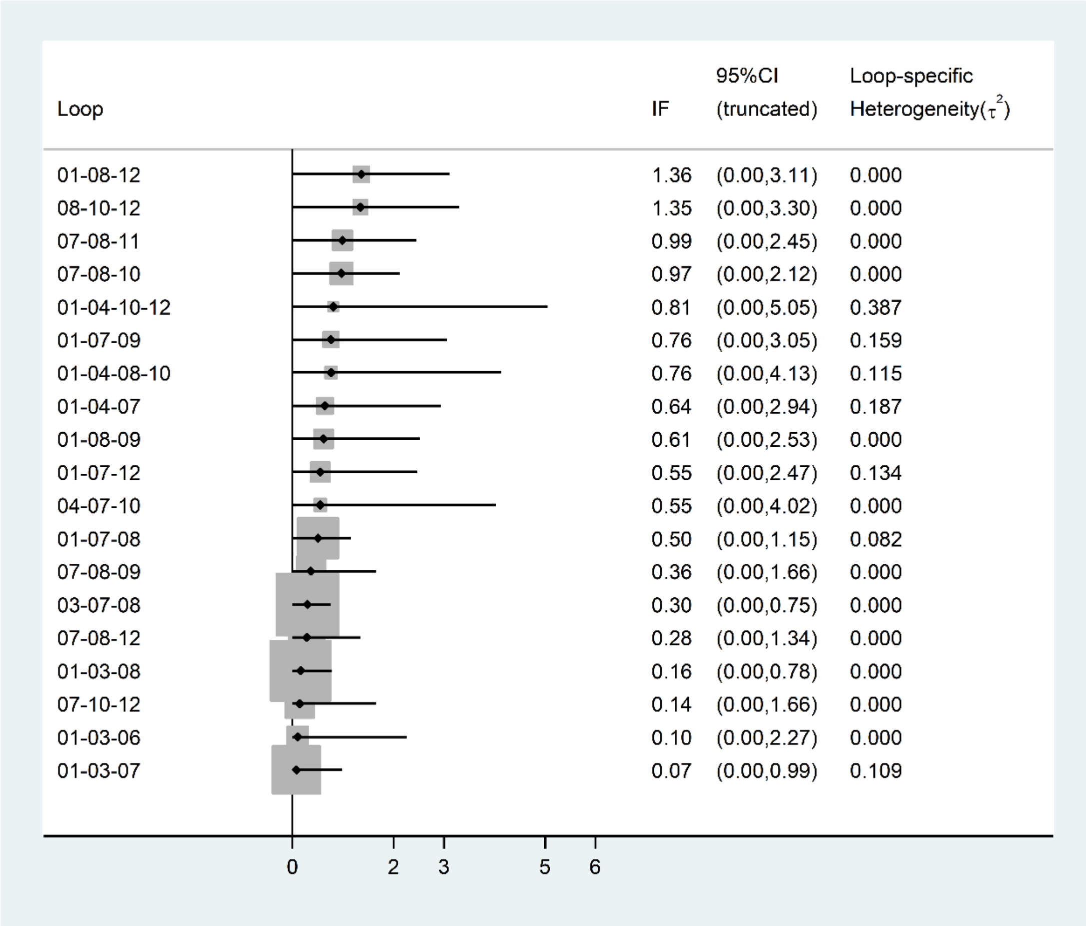

Legend: Inconsistency plot assuming loop-specific heterogeneity estimates using the method of moments estimator. arm 01= placebo; arm 02= oral polyene; arm 03= conventional amphotericin B (AMB); arm 04= liposomal amphotericin B (AMBL); arm 05= miconazole (MICZ); arm 06= ketoconazole (KTCZ); arm 07= fluconazole (FLCZ); arm 08= itraconazole (ITCZ); arm 09= voriconazole (VOCZ); arm 10= posaconazole (POCZ); arm 11= caspofungin (CASP); arm 12= micafungin (MCFG).

© 2020 Wang J et al. *JAMA Netw Open.*

Panel C. Proven IFI

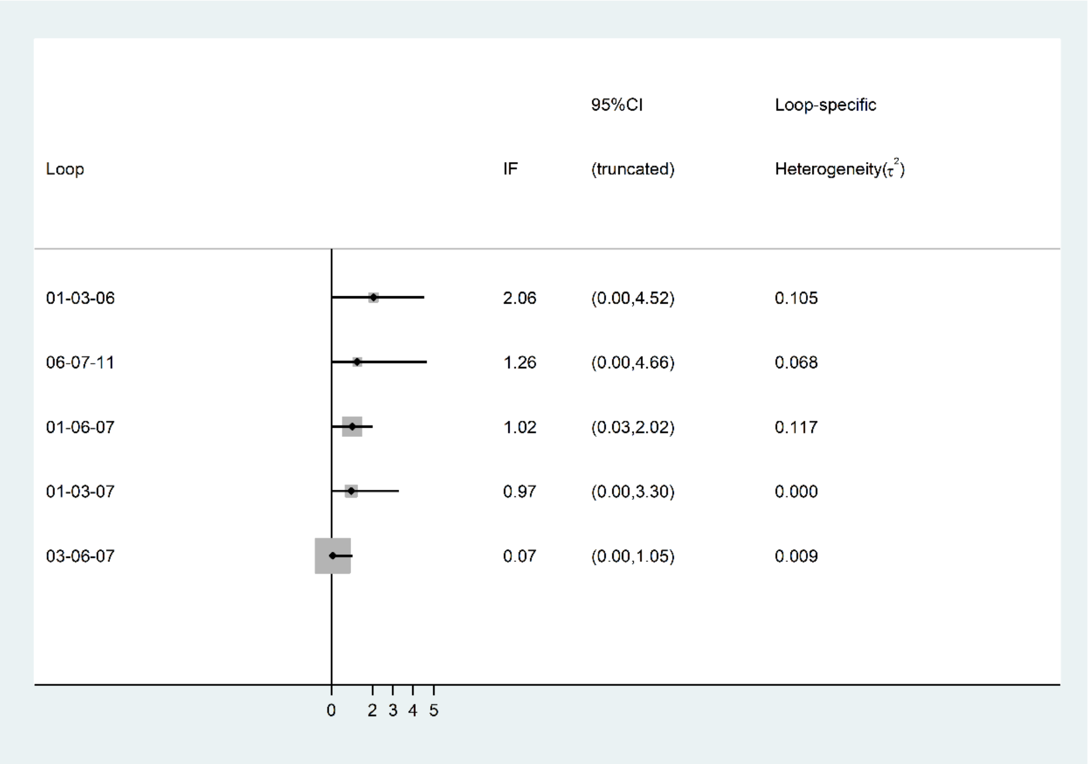

Legend: Inconsistency plot assuming loop-specific heterogeneity estimates using the method of moments estimator. arm 01= placebo; arm 02= oral polyene; arm 03= conventional amphotericin B (AMB); arm 04= liposomal amphotericin B (AMBL); arm 05= miconazole (MICZ); arm 06= ketoconazole (KTCZ); arm 07= fluconazole (FLCZ); arm 08= itraconazole (ITCZ); arm 09= voriconazole (VOCZ); arm 10= posaconazole (POCZ); arm 11= caspofungin (CASP); arm 12= micafungin (MCFG).

© 2020 Wang J et al. *JAMA Netw Open.*

Panel D. Invasive Candidiasis

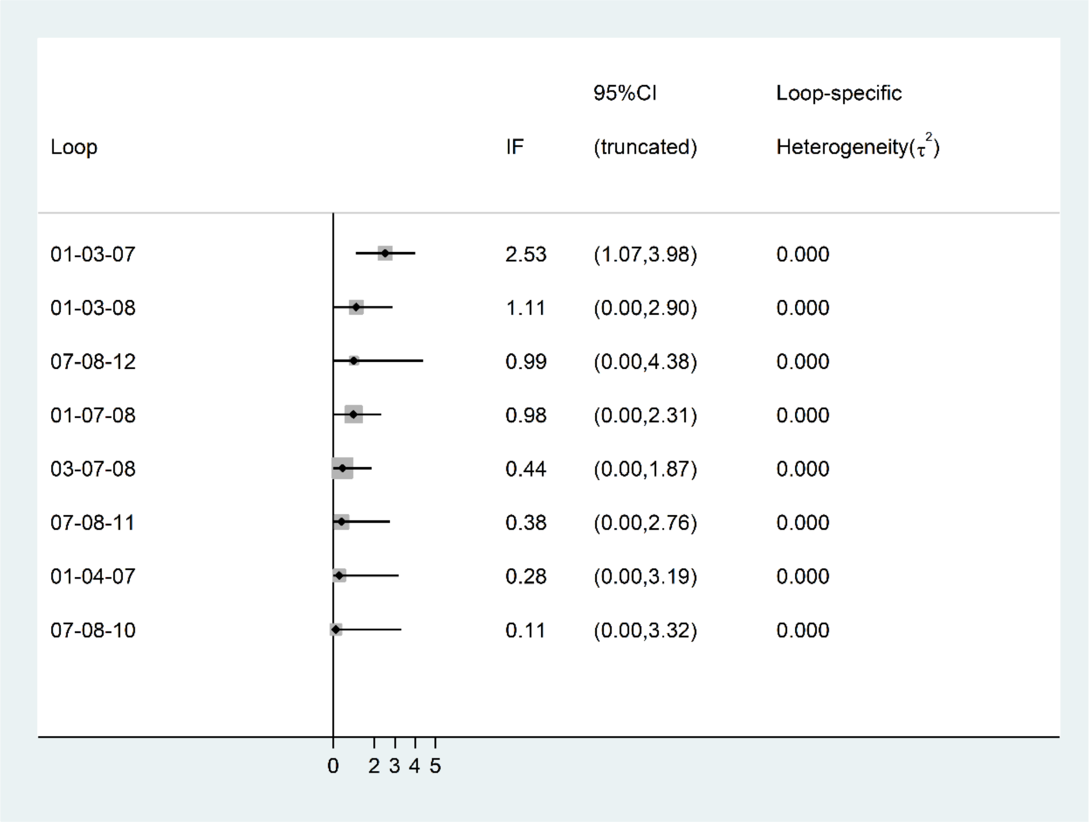

Legend: Inconsistency plot assuming loop-specific heterogeneity estimates using the method of moments estimator. arm 01= placebo; arm 02= oral polyene; arm 03= conventional amphotericin B (AMB); arm 04= liposomal amphotericin B (AMBL); arm 05= miconazole (MICZ); arm 06= ketoconazole (KTCZ); arm 07= fluconazole (FLCZ); arm 08= itraconazole (ITCZ); arm 09= voriconazole (VOCZ); arm 10= posaconazole (POCZ); arm 11= caspofungin (CASP); arm 12= micafungin (MCFG).

© 2020 Wang J et al. *JAMA Netw Open.*

Panel E. Invasive Aspergillus

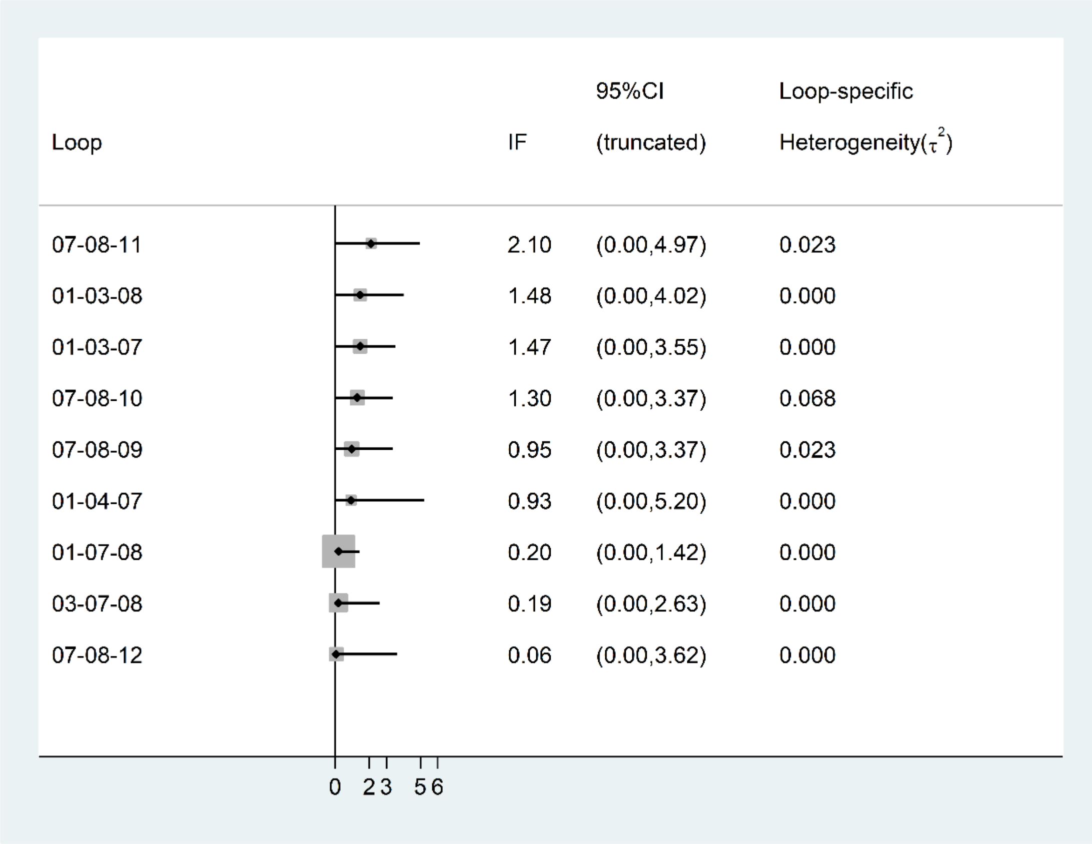

Legend: Inconsistency plot assuming loop-specific heterogeneity estimates using the method of moments estimator. arm 01= placebo; arm 02= oral polyene; arm 03= conventional amphotericin B (AMB); arm 04= liposomal amphotericin B (AMBL); arm 05= miconazole (MICZ); arm 06= ketoconazole (KTCZ); arm 07= fluconazole (FLCZ); arm 08= itraconazole (ITCZ); arm 09= voriconazole (VOCZ); arm 10= posaconazole (POCZ); arm 11= caspofungin (CASP); arm 12= micafungin (MCFG).

© 2020 Wang J et al. *JAMA Netw Open.*

Panel F. Mortality

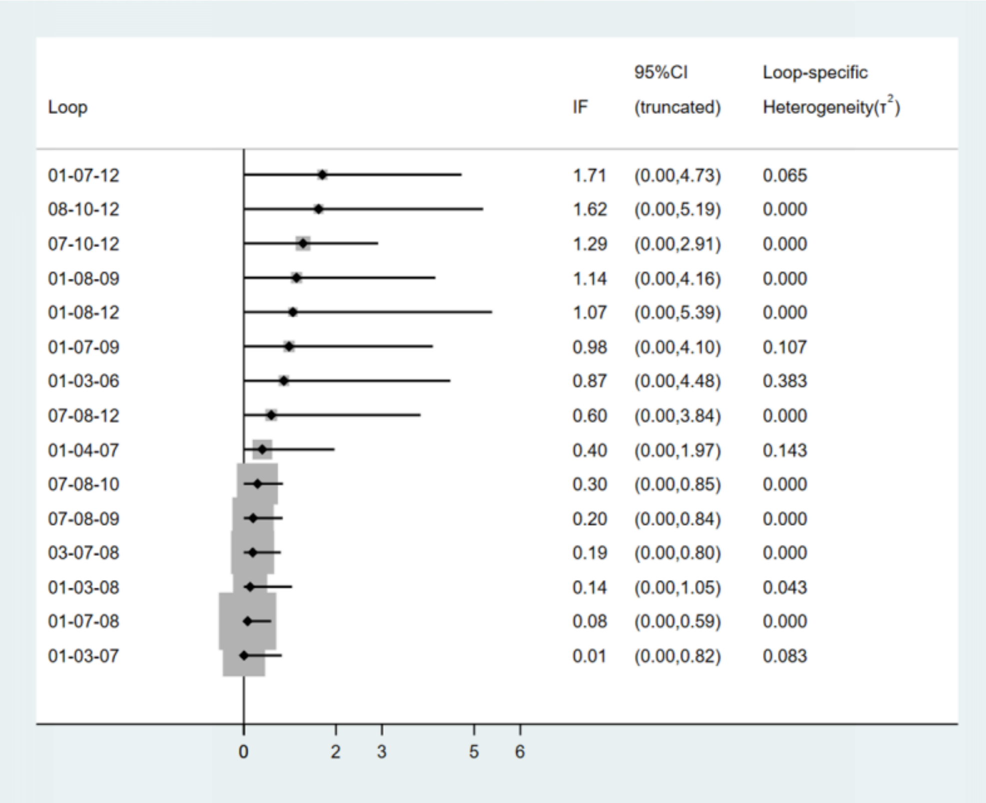

Legend: Inconsistency plot assuming loop-specific heterogeneity estimates using the method of moments estimator. arm 01= placebo; arm 02= oral polyene; arm 03= conventional amphotericin B (AMB); arm 04= liposomal amphotericin B (AMBL); arm 05= miconazole (MICZ); arm 06= ketoconazole (KTCZ); arm 07= fluconazole (FLCZ); arm 08= itraconazole (ITCZ); arm 09= voriconazole (VOCZ); arm 10= posaconazole (POCZ); arm 11= caspofungin (CASP); arm 12= micafungin (MCFG).

© 2020 Wang J et al. *JAMA Netw Open.*

Panel G. Fungi-related death

Loop	IF	95%CI (truncated)	Loop-specific Heterogeneity(τ^2)
01-03-06	1.72	(0.00,5.82)	0.000
07-08-10	1.43	(0.11,2.76)	0.000
07-10-12	1.33	(0.00,4.66)	0.000
03-07-08	0.81	(0.00,2.55)	0.331
01-04-07	0.74	(0.00,3.90)	0.084
01-03-07	0.71	(0.00,2.07)	0.000
01-08-10-12	0.34	(0.00,5.21)	0.144
01-07-08	0.21	(0.00,1.77)	0.368
01-03-08	0.13	(0.00,1.71)	0.003
01-07-12	0.01	(0.00,3.46)	0.000

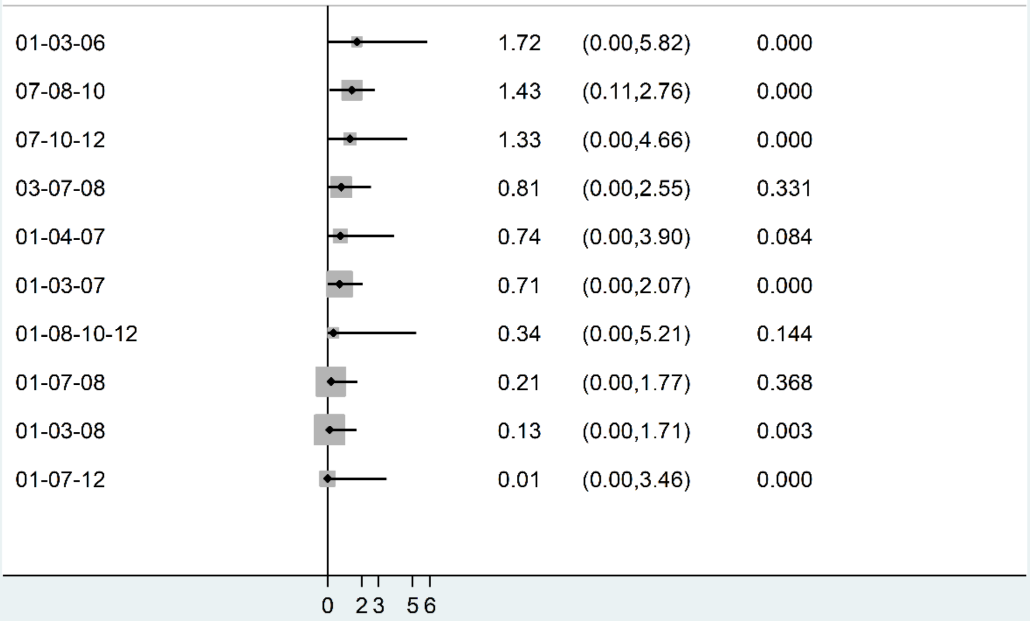

Legend: Inconsistency plot assuming loop-specific heterogeneity estimates using the method of moments estimator. arm 01= placebo; arm 02= oral polyene; arm 03= conventional amphotericin B (AMB); arm 04= liposomal amphotericin B (AMBL); arm 05= miconazole (MICZ); arm 06= ketoconazole (KTCZ); arm 07= fluconazole (FLCZ); arm 08= itraconazole (ITCZ); arm 09= voriconazole (VOCZ); arm 10= posaconazole (POCZ); arm 11= caspofungin (CASP); arm 12= micafungin (MCFG).

© 2020 Wang J et al. *JAMA Netw Open.*

Panel H. Withdrawal

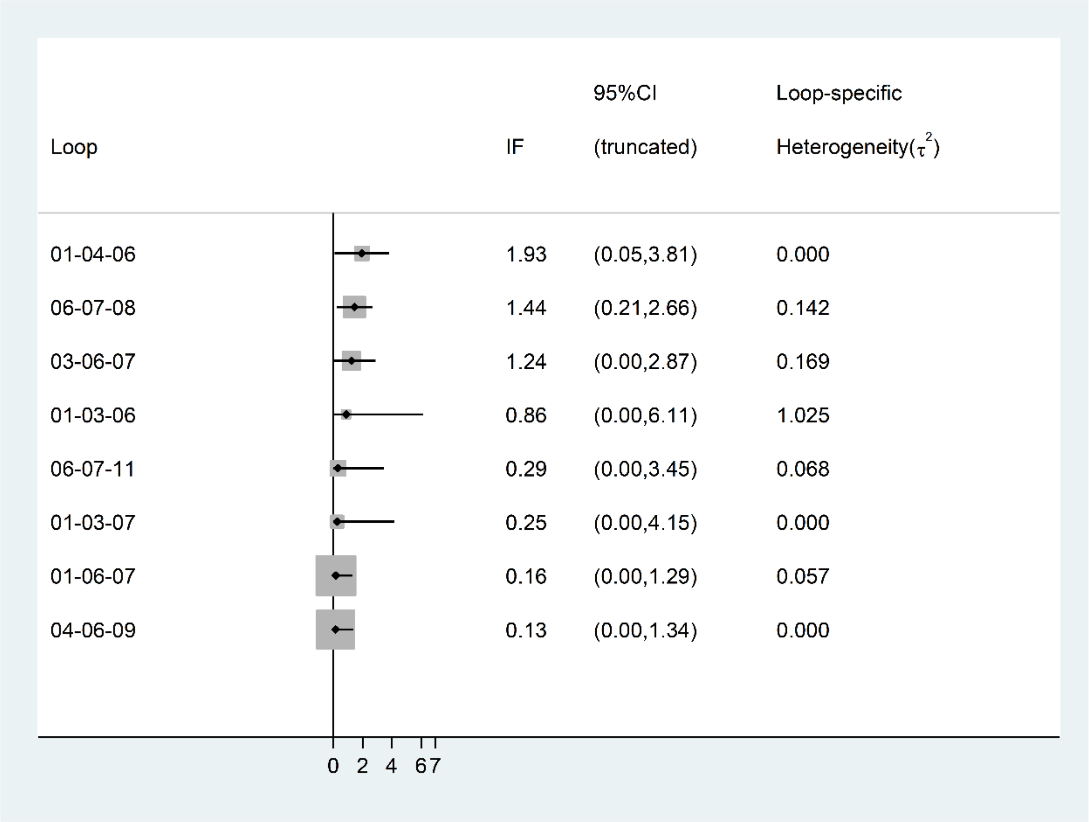

Legend: Inconsistency plot assuming loop-specific heterogeneity estimates using the method of moments estimator. arm 01= placebo; arm 02= oral polyene; arm 03= conventional amphotericin B (AMB); arm 04= liposomal amphotericin B (AMBL); arm 05= miconazole (MICZ); arm 06= ketoconazole (KTCZ); arm 07= fluconazole (FLCZ); arm 08= itraconazole (ITCZ); arm 09= voriconazole (VOCZ); arm 10= posaconazole (POCZ); arm 11= caspofungin (CASP); arm 12= micafungin (MCFG).

© 2020 Wang J et al. *JAMA Netw Open.*

eFigure 8. Funnel Plots of All Outcomes

Panel A. Fungal Infections

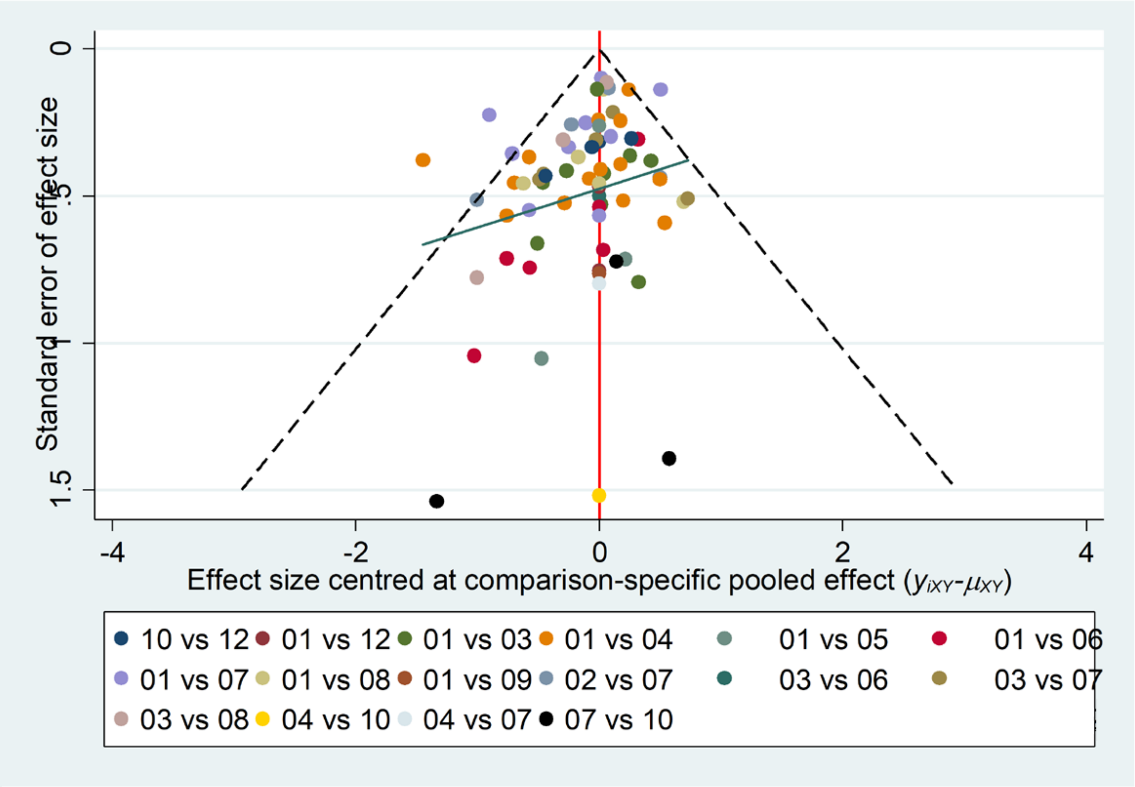

Legend: In the comparison-adjusted funnel plot, the horizontal axis shows the difference of each i-study estimate YiXY from the summary effect for the respective comparison (YiXY-µXY) while the vertical axis presents the measure of dispersion of YiXY, namely the standard error of the effect size. The red line shows the null hypothesis. Each point represents a direct comparison; different colors correspond to different comparisons. The dashed black line represents the 95% confidence interval. The horizontal line represents the regression line; the regression line demonstrates that no asymmetry is present. arm 01= placebo; arm 02= oral polyene; arm 03= conventional amphotericin B (AMB); arm 04= liposomal amphotericin B (AMBL); arm 05= miconazole (MICZ); arm 06= ketoconazole (KTCZ); arm 07= fluconazole (FLCZ); arm 08= itraconazole (ITCZ); arm 09= voriconazole (VOCZ); arm 10= posaconazole (POCZ); arm 11= caspofungin (CASP); arm 12= micafungin (MCFG).

© 2020 Wang J et al. *JAMA Netw Open.*

Panel B. IFI

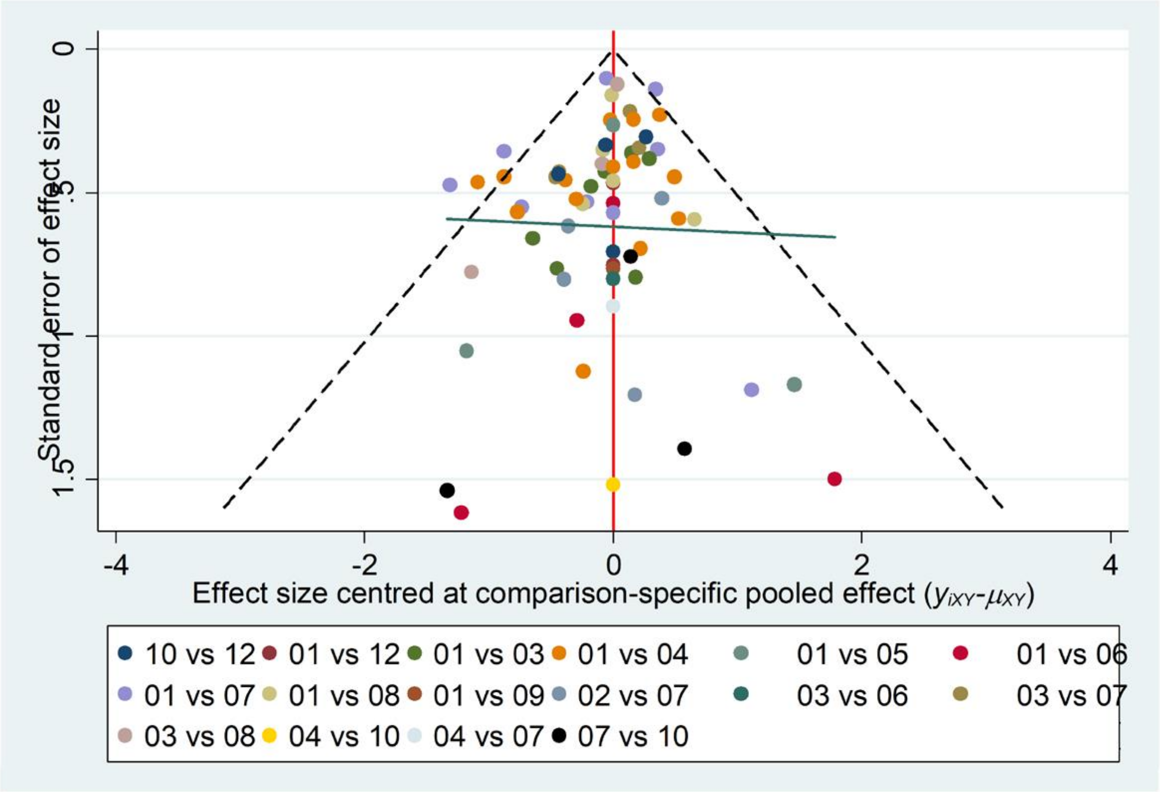

Legend: In the comparison-adjusted funnel plot, the horizontal axis shows the difference of each i-study estimate YiXY from the summary effect for the respective comparison (YiXY-µXY) while the vertical axis presents the measure of dispersion of YiXY, namely the standard error of the effect size. The red line shows the null hypothesis. Each point represents a direct comparison; different colors correspond to different comparisons. The dashed black line represents the 95% confidence interval. The horizontal line represents the regression line; the regression line demonstrates that no asymmetry is present. arm 01= placebo; arm 02= oral polyene; arm 03= conventional amphotericin B (AMB); arm 04= liposomal amphotericin B (AMBL); arm 05= miconazole (MICZ); arm 06= ketoconazole (KTCZ); arm 07= fluconazole (FLCZ); arm 08= itraconazole (ITCZ); arm 09= voriconazole (VOCZ); arm 10= posaconazole (POCZ); arm 11= caspofungin (CASP); arm 12= micafungin (MCFG).

© 2020 Wang J et al. *JAMA Netw Open.*

Panel C. Proven IFI

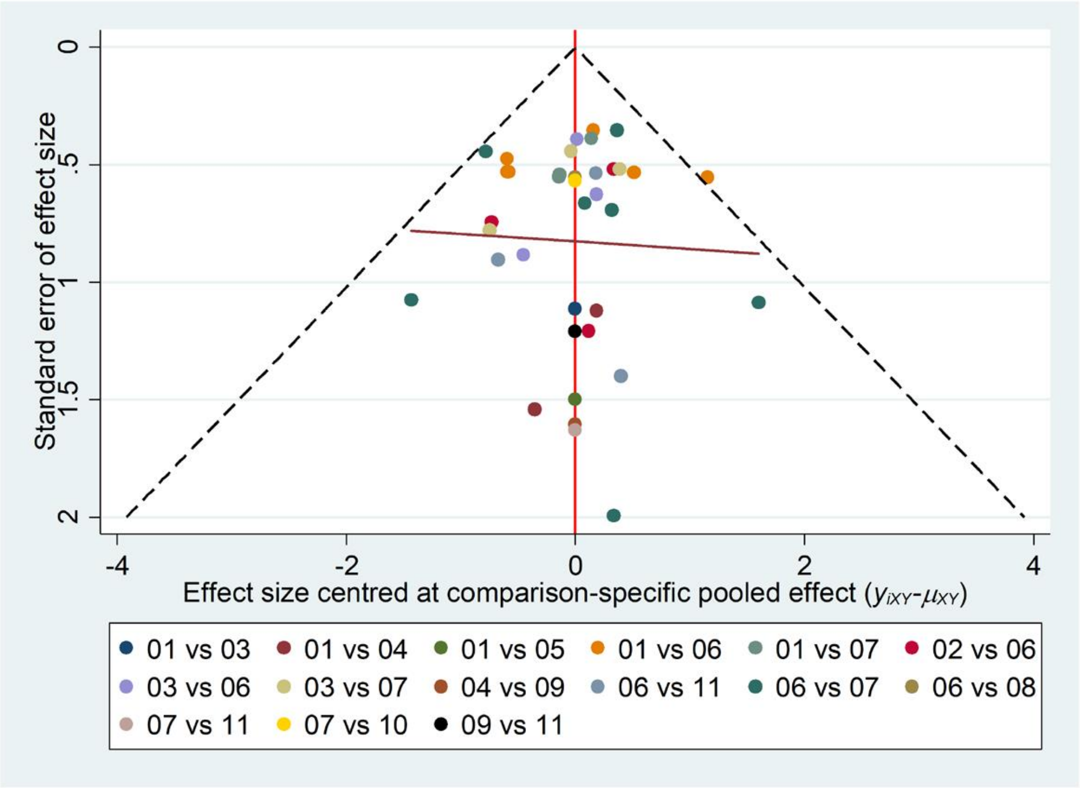

Legend: In the comparison-adjusted funnel plot, the horizontal axis shows the difference of each i-study estimate YiXY from the summary effect for the respective comparison (YiXY-µXY) while the vertical axis presents the measure of dispersion of YiXY, namely the standard error of the effect size. The red line shows the null hypothesis. Each point represents a direct comparison; different colors correspond to different comparisons. The dashed black line represents the 95% confidence interval. The horizontal line represents the regression line; the regression line demonstrates that no asymmetry is present. arm 01= placebo; arm 02= oral polyene; arm 03= conventional amphotericin B (AMB); arm 04= liposomal amphotericin B (AMBL); arm 05= miconazole (MICZ); arm 06= ketoconazole (KTCZ); arm 07= fluconazole (FLCZ); arm 08= itraconazole (ITCZ); arm 09= voriconazole (VOCZ); arm 10= posaconazole (POCZ); arm 11= caspofungin (CASP); arm 12= micafungin (MCFG).

© 2020 Wang J et al. *JAMA Netw Open.*

Panel D. Invasive Candidiasis

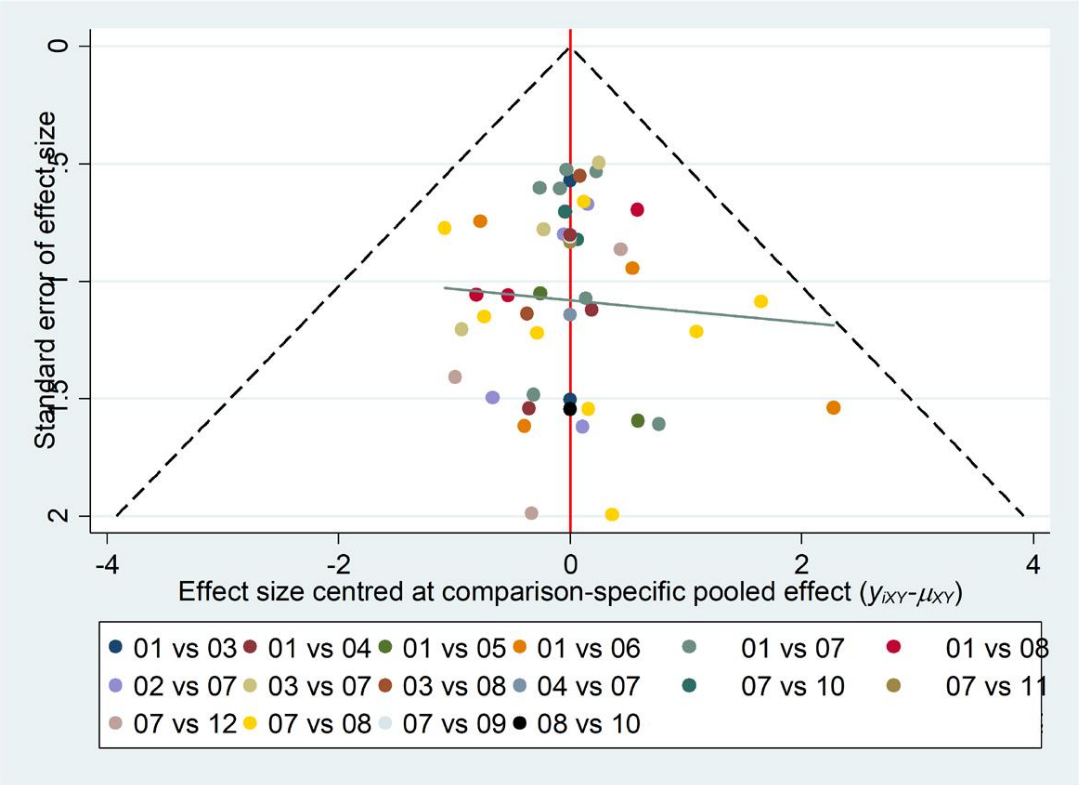

Legend: In the comparison-adjusted funnel plot, the horizontal axis shows the difference of each i-study estimate YiXY from the summary effect for the respective comparison (YiXY-µXY) while the vertical axis presents the measure of dispersion of YiXY, namely the standard error of the effect size. The red line shows the null hypothesis. Each point represents a direct comparison; different colors correspond to different comparisons. The dashed black line represents the 95% confidence interval. The horizontal line represents the regression line; the regression line demonstrates that no asymmetry is present. arm 01= placebo; arm 02= oral polyene; arm 03= conventional amphotericin B (AMB); arm 04= liposomal amphotericin B (AMBL); arm 05= miconazole (MICZ); arm 06= ketoconazole (KTCZ); arm 07= fluconazole (FLCZ); arm 08= itraconazole (ITCZ); arm 09= voriconazole (VOCZ); arm 10= posaconazole (POCZ); arm 11= caspofungin (CASP); arm 12= micafungin (MCFG).

© 2020 Wang J et al. *JAMA Netw Open.*

Panel E. Invasive Aspergillus

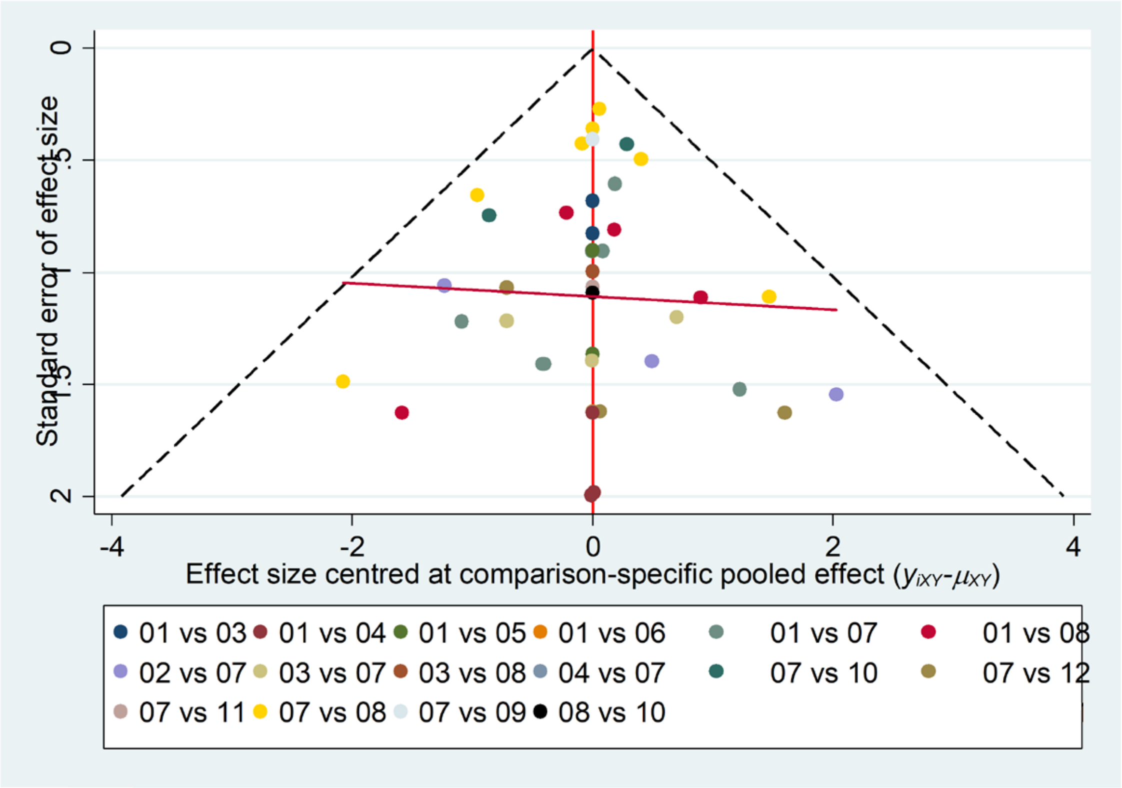

Legend: In the comparison-adjusted funnel plot, the horizontal axis shows the difference of each i-study estimate YiXY from the summary effect for the respective comparison (YiXY-µXY) while the vertical axis presents the measure of dispersion of YiXY, namely the standard error of the effect size. The red line shows the null hypothesis. Each point represents a direct comparison; different colors correspond to different comparisons. The dashed black line represents the 95% confidence interval. The horizontal line represents the regression line; the regression line demonstrates that no asymmetry is present. arm 01= placebo; arm 02= oral polyene; arm 03= conventional amphotericin B (AMB); arm 04= liposomal amphotericin B (AMBL); arm 05= miconazole (MICZ); arm 06= ketoconazole (KTCZ); arm 07= fluconazole (FLCZ); arm 08= itraconazole (ITCZ); arm 09= voriconazole (VOCZ); arm 10= posaconazole (POCZ); arm 11= caspofungin (CASP); arm 12= micafungin (MCFG).

© 2020 Wang J et al. *JAMA Netw Open.*

Panel F. Mortality

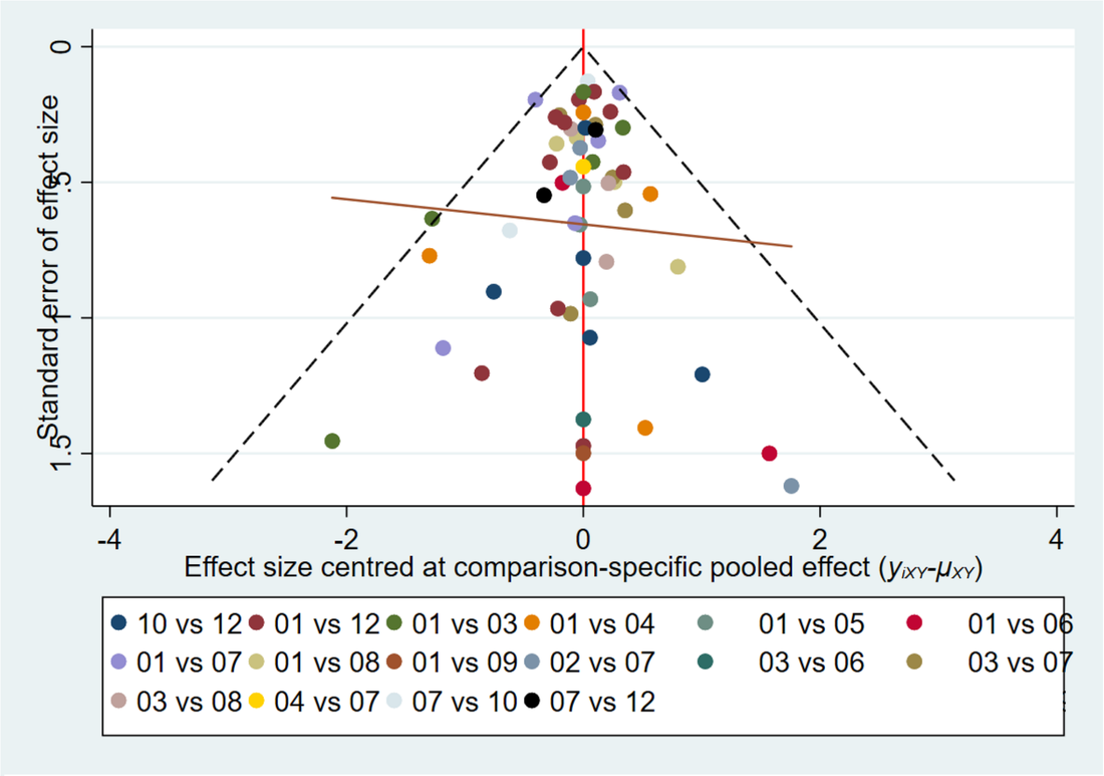

Legend: In the comparison-adjusted funnel plot, the horizontal axis shows the difference of each i-study estimate YiXY from the summary effect for the respective comparison (YiXY-µXY) while the vertical axis presents the measure of dispersion of YiXY, namely the standard error of the effect size. The red line shows the null hypothesis. Each point represents a direct comparison; different colors correspond to different comparisons. The dashed black line represents the 95% confidence interval. The horizontal line represents the regression line; the regression line demonstrates that no asymmetry is present. arm 01= placebo; arm 02= oral polyene; arm 03= conventional amphotericin B (AMB); arm 04= liposomal amphotericin B (AMBL); arm 05= miconazole (MICZ); arm 06= ketoconazole (KTCZ); arm 07= fluconazole (FLCZ); arm 08= itraconazole (ITCZ); arm 09= voriconazole (VOCZ); arm 10= posaconazole (POCZ); arm 11= caspofungin (CASP); arm 12= micafungin (MCFG).

© 2020 Wang J et al. *JAMA Netw Open.*

Panel G. Fungi-related death

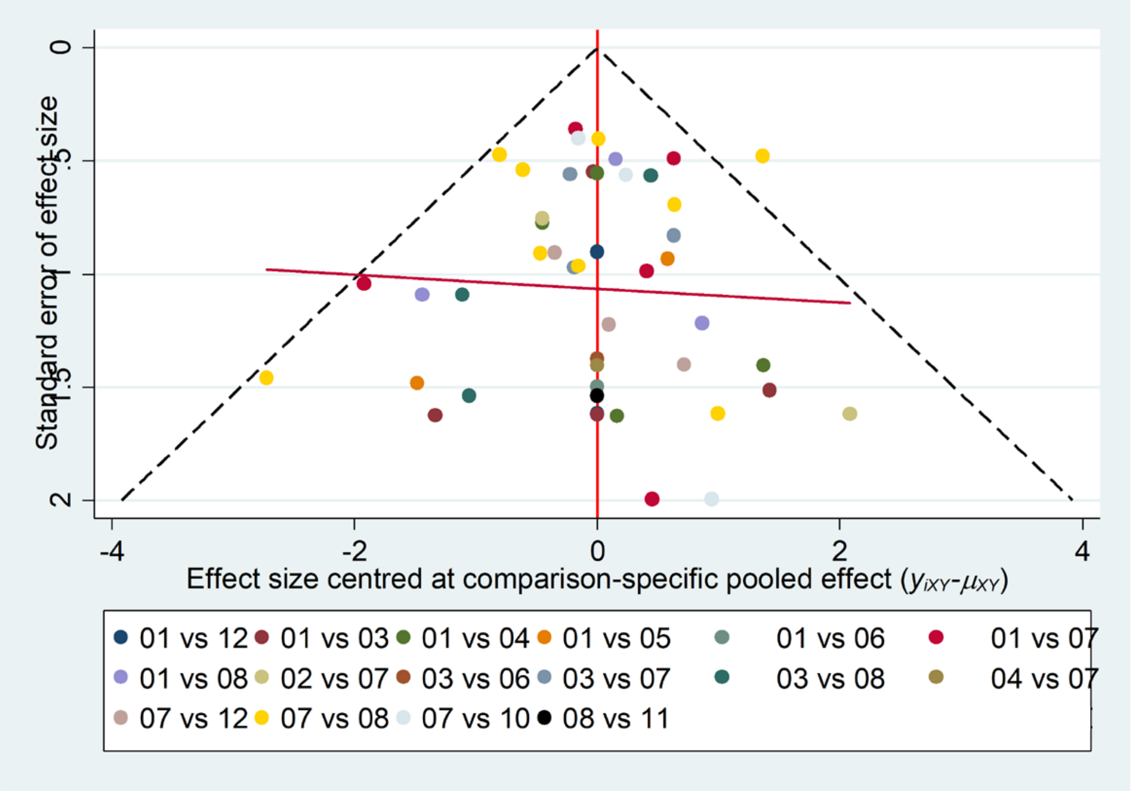

Legend: In the comparison-adjusted funnel plot, the horizontal axis shows the difference of each i-study estimate YiXY from the summary effect for the respective comparison (YiXY-µXY) while the vertical axis presents the measure of dispersion of YiXY, namely the standard error of the effect size. The red line shows the null hypothesis. Each point represents a direct comparison; different colors correspond to different comparisons. The dashed black line represents the 95% confidence interval. The horizontal line represents the regression line; the regression line demonstrates that no asymmetry is present. arm 01= placebo; arm 02= oral polyene; arm 03= conventional amphotericin B (AMB); arm 04= liposomal amphotericin B (AMBL); arm 05= miconazole (MICZ); arm 06= ketoconazole (KTCZ); arm 07= fluconazole (FLCZ); arm 08= itraconazole (ITCZ); arm 09= voriconazole (VOCZ); arm 10= posaconazole (POCZ); arm 11= caspofungin (CASP); arm 12= micafungin (MCFG).

© 2020 Wang J et al. *JAMA Netw Open.*

Panel H. Withdrawal

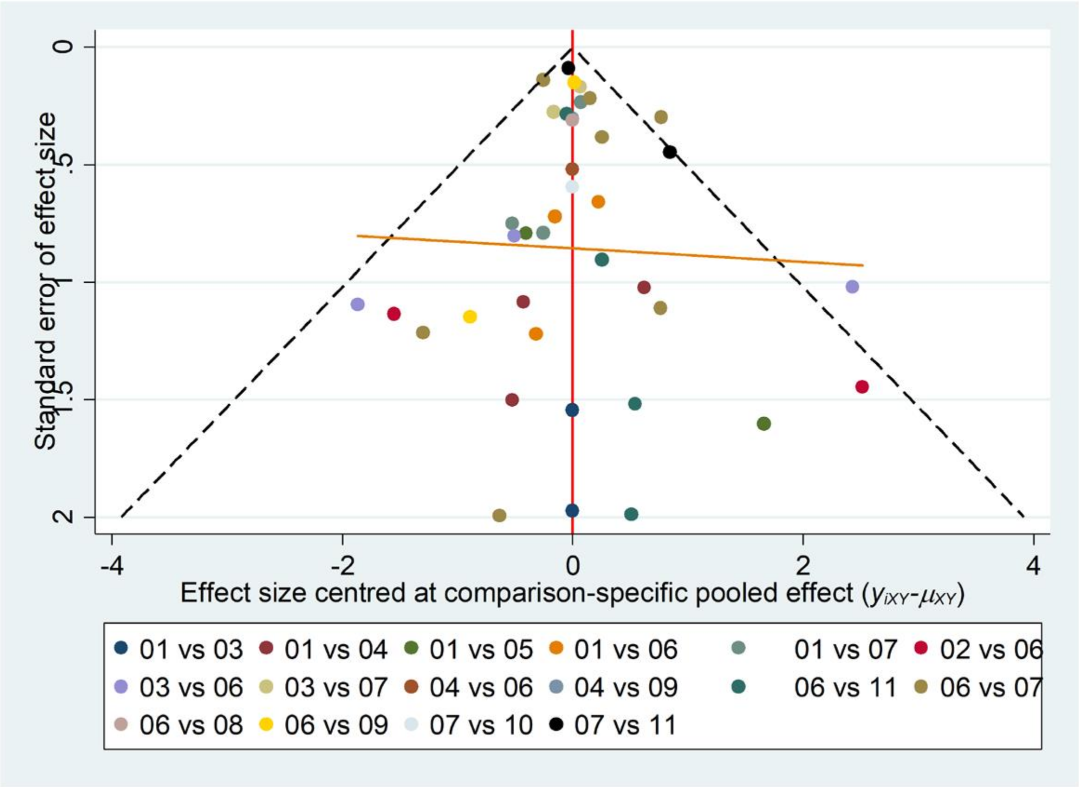

Legend: In the comparison-adjusted funnel plot, the horizontal axis shows the difference of each i-study estimate YiXY from the summary effect for the respective comparison (YiXY-µXY) while the vertical axis presents the measure of dispersion of YiXY, namely the standard error of the effect size. The red line shows the null hypothesis. Each point represents a direct comparison; different colors correspond to different comparisons. The dashed black line represents the 95% confidence interval. The horizontal line represents the regression line; the regression line demonstrates that no asymmetry is present. arm 01= placebo; arm 02= oral polyene; arm 03= conventional amphotericin B (AMB); arm 04= liposomal amphotericin B (AMBL); arm 05= miconazole (MICZ); arm 06= ketoconazole (KTCZ); arm 07= fluconazole (FLCZ); arm 08= itraconazole (ITCZ); arm 09= voriconazole (VOCZ); arm 10= posaconazole (POCZ); arm 11= caspofungin (CASP); arm 12= micafungin (MCFG).

© 2020 Wang J et al. *JAMA Netw Open.*
